# Transcriptome analysis of *Cinnamomum migao* seed germination in medicinal plants of Southwest China

**DOI:** 10.1186/s12870-021-03020-7

**Published:** 2021-06-11

**Authors:** Xiaolong Huang, Tian Tian, Jingzhong Chen, Deng Wang, Bingli Tong, Jiming Liu

**Affiliations:** 1 Department of Ecology, College of Forestry, Guizhou University, 550025 Guiyang, China; 2 Forest Ecology Research Center of Guizhou University, 550025 Guiyang, China; 3 Key laboratory of Plant Resource Conservation and Germplasm Innovation in Mountainous Region (Ministry of Education), Collaborative Innovation Center for Mountain Ecology & Agro-Bioengineering (CICMEAB), Institute of Agro-bioengineering/College of Life Sciences, Guizhou University, 550025 Guiyang, China

**Keywords:** Seed germination, Transcriptome, Material metabolism, Metabolic network

## Abstract

**Background:**

*Cinnamomum migao* is an endangered evergreen woody plant species endemic to China. Its fruit is used as a traditional medicine by the Miao nationality of China and has a high commercial value. However, its seed germination rate is extremely low under natural and artificial conditions. As the foundation of plant propagation, seed germination involves a series of physiological, cellular, and molecular changes; however, the molecular events and systematic changes occurring during *C. migao* seed germination remain unclear.

**Results:**

In this study, combined with the changes in physiological indexes and transcription levels, we revealed the regulation characteristics of cell structures, storage substances, and antioxidant capacity during seed germination. Electron microscopy analysis revealed that abundant smooth and full oil bodies were present in the cotyledons of the seeds. With seed germination, oil bodies and other substances gradually degraded to supply energy; this was consistent with the content of storage substances. In parallel to electron microscopy and physiological analyses, transcriptome analysis showed that 80–90 % of differentially expressed genes (DEGs) appeared after seed imbibition, reflecting important development and physiological changes. The unigenes involved in material metabolism (glycerolipid metabolism, fatty acid degradation, and starch and sucrose metabolism) and energy supply pathways (pentose phosphate pathway, glycolysis pathway, pyruvate metabolism, tricarboxylic acid cycle, and oxidative phosphorylation) were differentially expressed in the four germination stages. Among these DEGs, a small number of genes in the energy supply pathway at the initial stage of germination maintained high level of expression to maintain seed vigor and germination ability. Genes involved in lipid metabolism were firstly activated at a large scale in the LK (seed coat fissure) stage, and then genes involved in carbohydrates (CHO) metabolism were activated, which had their own species specificity.

**Conclusions:**

Our study revealed the transcriptional levels of genes and the sequence of their corresponding metabolic pathways during seed germination. The changes in cell structure and physiological indexes also confirmed these events. Our findings provide a foundation for determining the molecular mechanisms underlying seed germination.

**Supplementary Information:**

The online version contains supplementary material available at 10.1186/s12870-021-03020-7.

## Background

*Cinnamomum migao* H. W. Li is a species of the most important evergreen medicinal trees of the family Lauraceae. As a native plant, it is primarily distributed in Southwest China [1] and is defined as an endangered species by the Red Paper of Endangered Plants in China. In addition, owing to its special efficacy for angiocardiopathy and stomachache, *C. migao* has been commended as a famous and genuine medicinal material in Guizhou province. However, excessive utilization of wild resources and the low germination rate of *C. migao* have resulted in the rare regeneration of individuals in the natural environment. Furthermore, previous studies and our previous research field investigations have found that the number of the species has decreased [2, 3].

Seed germination is considered a prerequisite for the establishment of plant seedlings and is a crucial stage in the life cycle of species [4, 5]. The process of seed germination begins with water imbibition by mature dry seeds and ends with radicle protrusion. This processes are regulated by the coordination of several complex physiological, biochemical, and molecular processes [6], including the mobilization of stored reserves, energy production, signaling transduction, and transcription activation [7]. As a rule, there are three important phases that seeds have to undergo to complete the final germination from the static state of maturity. After the initial rapid uptake (Phase I), the absorption of water into cells promotes the rapid expansion of dry seeds. Next, the physiological and metabolic processes of seeds begin, triggering the mobilization of internal storage substances, such as proteins, lipids and starch [8]. Subsequently, seed enter a relatively plateau stage (Phase II), wherein its internal metabolism is further activated, resulting in the synthesis of a large number of mitochondrial [9], repair and *de novo* synthesis of DNA, mobilization and degradation of storage proteins, and translation of storage mRNA [10]. Seed germination enters the third stage (Phase III) with the elongation of embryonic axes and the growth of radicle, which is usually described as the post-germination stage [11, 12]. During seed germination, energy production and respiration play vital roles [13], and the initial energy produced is mainly provided via anaerobic respiration. Subsequently, respiratory activity increases as oxygen uptake and carbon dioxide release accelerate during water uptake [14]. The physiological process of seed germination requires considerable energy [15]. The main energy supply of cellular metabolism comes from glucose, which is catabolized by subsequent processes, i.e., glycolysis and the tricarboxylic acid (TCA) cycle, and finally by oxidative phosphorylation to produce ATP.

Seed germination rate has a high correlation with the survival rate of seedlings and subsequent growth and development of seedlings, which directly affects the quality of seedlings [16]. Physiological and biochemical studies were the main concerns of preliminary studies on seeds germination [17]; these studies allowed us to obtain a basic understanding of the major physiological changes and reactivation of metabolic processes that occur during seed germination [5, 18]. Transcriptional sequencing is an effective tool to understand the complex molecular regulatory mechanisms as well as to provide new insights into seed germination. This technique has been performed during the seed germination of several plants, including *Hordeum vulgare*, *Brassica napus*, *Suaeda aralocaspica*, and *Zanthoxylum dissitum* [19–22]; however, the molecular mechanisms underlying the seed germination of woody plant still remain elusive, particularly, those of medicinal plants [23]. Therefore, the RNA-Seq technology was the technique used to investigate the molecular processes of *C. migao* of Lauraceae for providing valuable insights into seed germination in medicinal plants.

## Results

### Morphology and scanning electron microscopy observation during *C. migao* seed germination

**Fig. 1:**
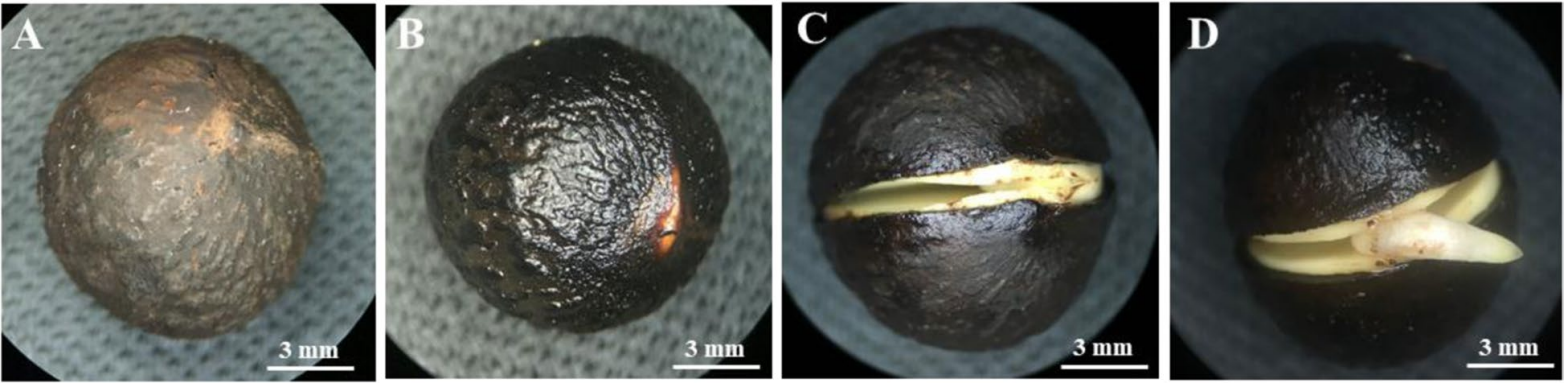
Morphological characteristics of *Cinnamomum migao* seeds in the four stages of germination. **A**. Dry seeds (seed without imbibition, GZ). **B**. 72 h after imbibition (seeds imbibed water to full imbibition, XS). **C**. Seed dehiscence stage (seed coat fissure after imbibition for 24 days, LK). **D**. Seed germination stage (radicle protruding the seed coat 4 mm after imbibition for 31 days, MF)

**Fig. 2:**
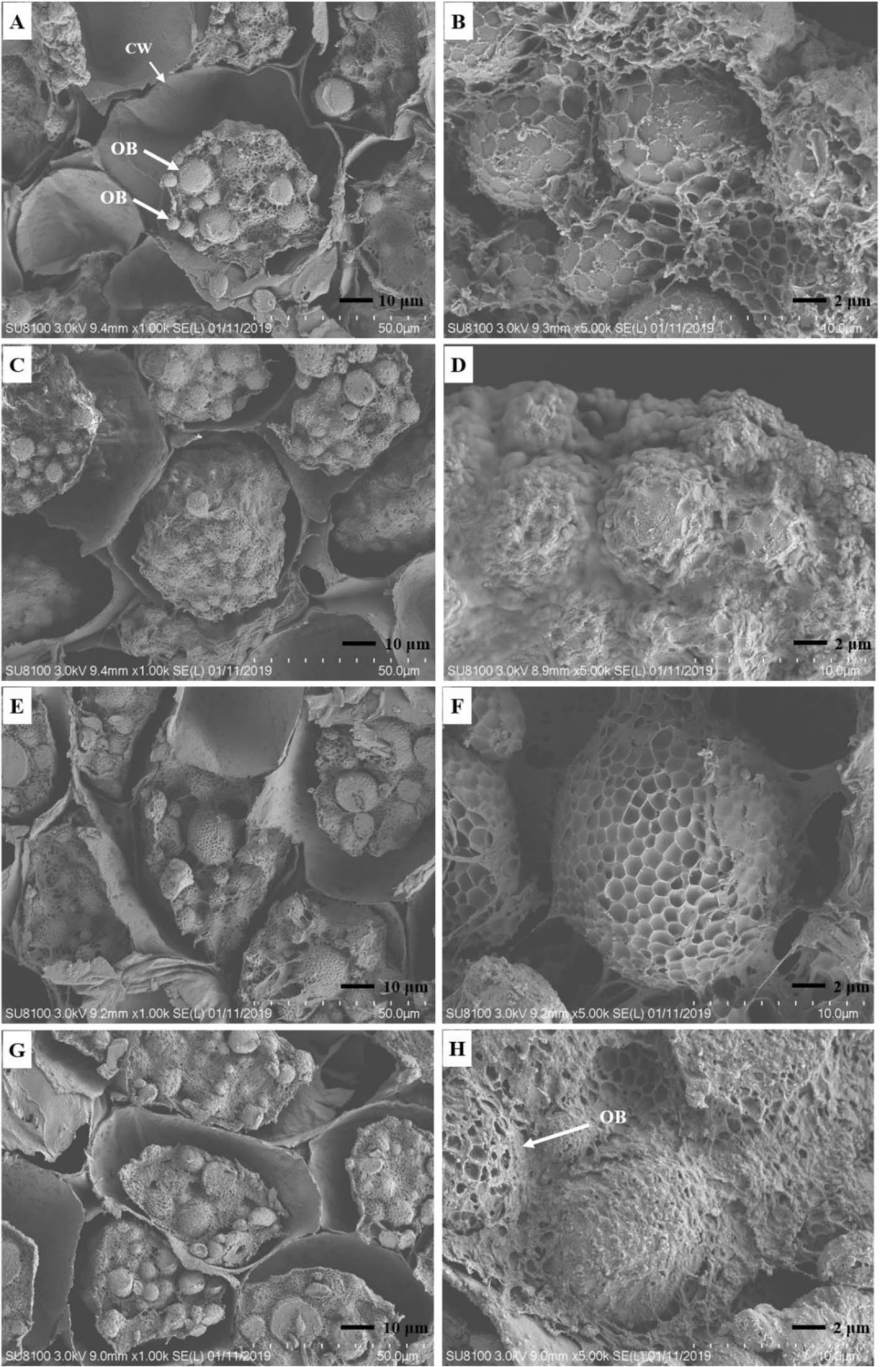
Scanning electron microscopic images of *Cinnamomum migao* seed germination. **A**, **B**. Seeds without water absorption. **C**, **D**. Seeds imbibed water for 72 h until full imbibition. **E**, **F**. Seeds coat fissure. **G**, **H**. Radicle protruding seed coat (4 mm). OB: oil body. CW: cell wall

During the four germination stages of *C. migao* seeds (Fig. 1), structural changes were observed in the cotyledon cells under a scanning electron microscope (Fig. 2). The diameter of the cotyledon cells were 52–72 μm in the cell full of oil bodies. In the dry seed stage, smooth and plump spherical oil bodies with a diameter of 3–12 μm were present in the cotyledon cells. Globular particles, such as oil bodies, were covered with amorphous enzyme (Fig. 2 A and B). With seed germination, tangible microstructural changes could be observed in the oil bodies and other storage substances in cells under the action of enzymes. After the seeds absorbed water, the oil bodies were completely wrapped by the activated amorphous enzyme, the structure of the smooth oil bodies disappeared (Fig. 2 C and D), and some precipitates were observed on oil bodies, making their surfaces rough. From the seed dehiscence to germination stage, the surface of the oil bodies were hollow and honeycomb and the oil bodies in the cell were significantly consumed (Fig. 2E–H).

### Physiological characteristics of seed germination

**Fig. 3:**
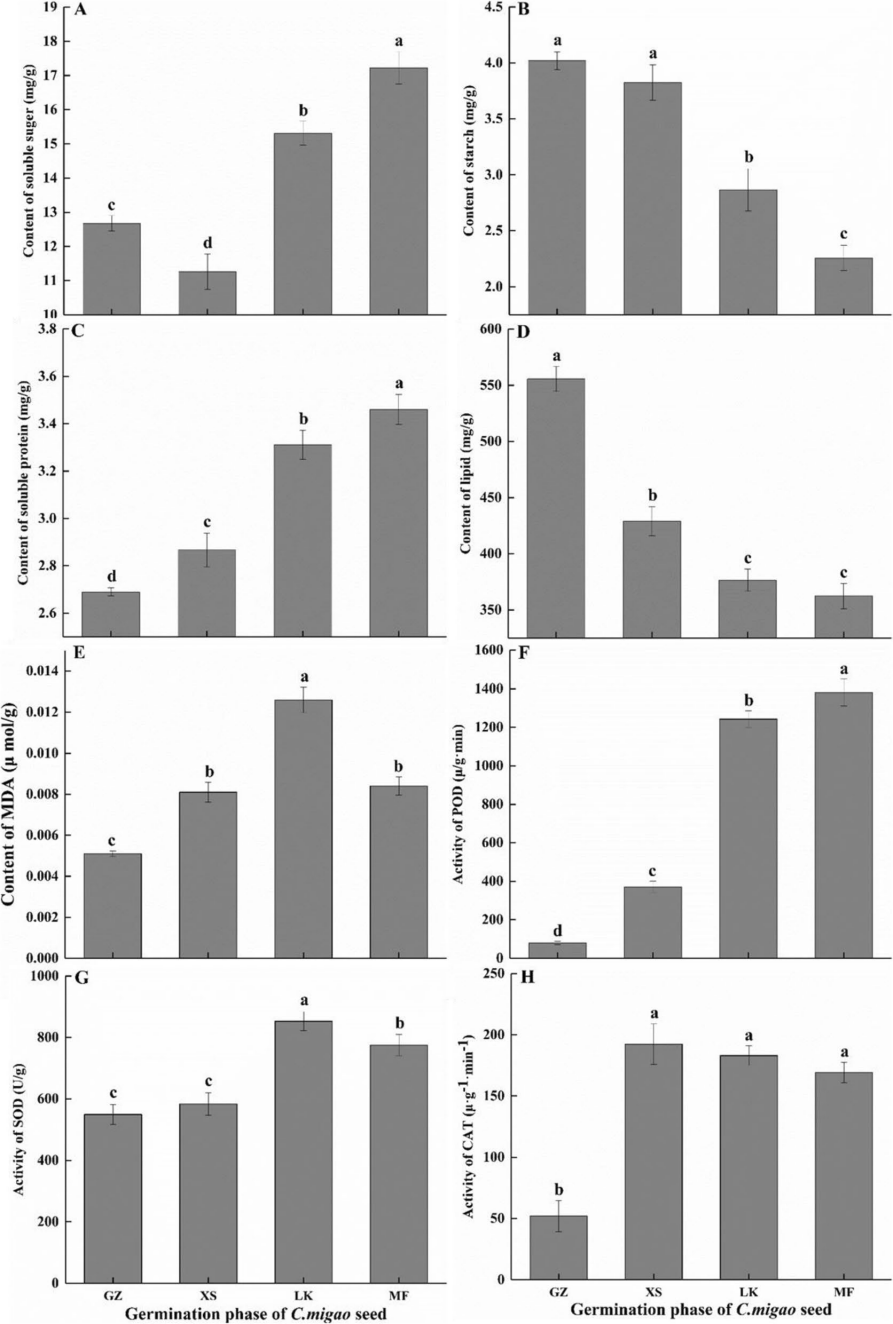
Changes in the storage substances during the different seed germination phases of *Cinnamomum migao*. **A**. Content of soluble suger. **B**. Content of starch. **C**. Content of soluble protein. **D**. Content of lipid. **E**. Content of MDA (malondialdehyde). **F**. Activity of POD (peroxidase). **G**. Activity of SOD (superoxide dismutase). **H**. Activity of CAT (catalase). The x-axis denotes the different stages of seed germination and the y-axis denotes the content of the storage substances and MDA as well as the activities of antioxidative enzymes. Values are expressed as mean ± standard deviation (n = 3). The values marked with different letters are significant at *p* < 0.05

Compared with dry seeds, the soluble sugar content (SSC) and starch and lipid contents of unit weight seed significantly decreased with the rapid entry of water, which decreased by 11.06 %, 4.87 %, and 32.25 %, respectively; conversely, the soluble protein content significantly increased by 6.59 %. Compared with dry seeds, the SSC and soluble protein content continuously increased in the seeds in the dehiscence and germination stages, with the highest increase of 35.88 and 28.66 %, respectively; the starch and lipid content of storage materials decreased during seed germination by 28.69 and 43.86 % and 32.25 and 34.79 %, respectively (Fig. 3).

### Transcriptome functional annotation and expression profiling

**Fig. 4:**
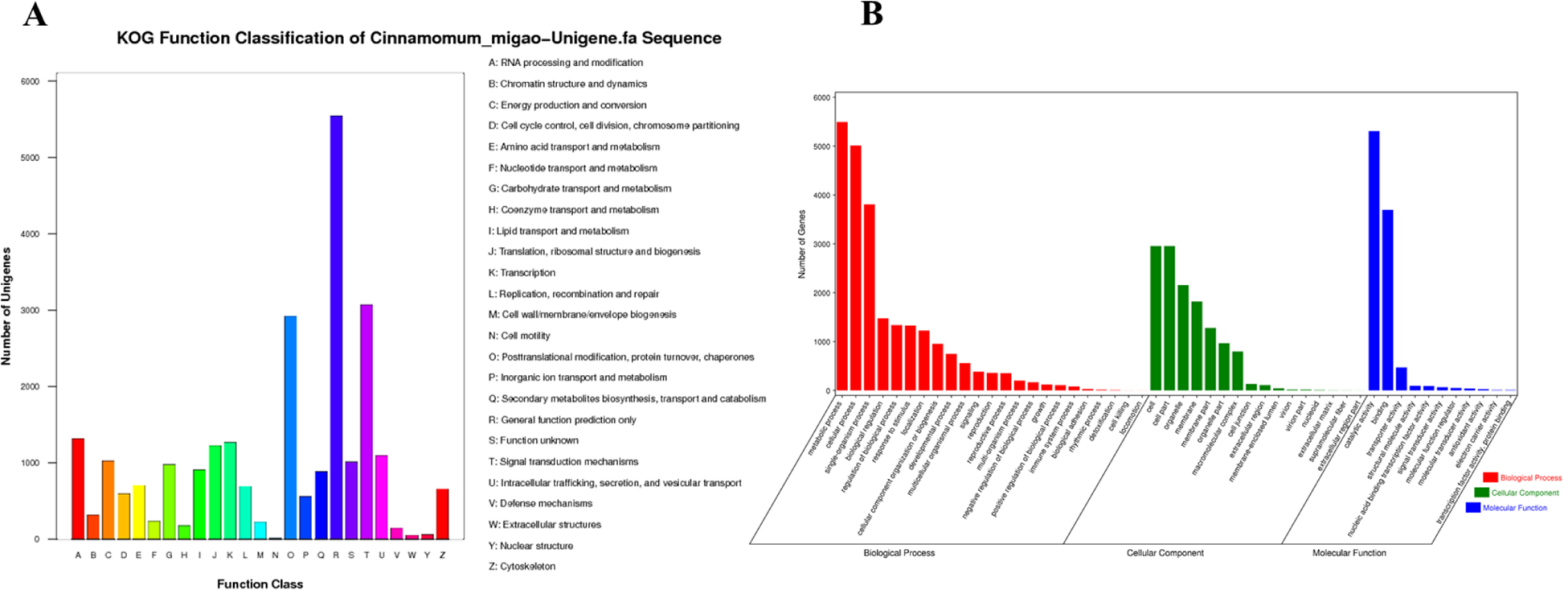
Functional annotations of the unigenes of the *Cinnamomum migao* seed transcriptome. **A**. KOG functional annotation of *Cinnamomum migao* seeds. **B**. GO functional annotation of *Cinnamomum migao* seeds

With the aim of investigating the transcriptional landscape of *C. migao* seed germination, we performed RNA-Seq to analyze the variations in transcript levels during the four stages of germination (each stage sample contained three replicates). The raw data were filtered from 12 cDNA libraries, and the Q30 of all samples were > 95 % (Table S1). Assembling of the sequencing data led to the identification of a total of 78,832 unigenes; the length of N50 was 1,560 bp. To obtain comprehensive information on the assembled transcriptomes, the nonredundant sequences were annotated based on a similarity search against the Nr, Swissprot, KOG, KEGG, and GO databases using a significant threshold E-value of ≤ 10^− 5^ and the BLAST algorithm. In addition, 78,832 unigenes distributed to each of the databases, with 31,313 (39.72 % of the total) for Nr, 17,047 (21.62 %) for KOG, 19,363 (24.56 %) for SwissProt, 12,305 (15.61 %) for KEGG, and 9,284 (11.78 %) for GO were investigated (Fig. 4). Unfortunately, 47,302 unigenes (60.0 %) could not be functionally annotated in the current study, which was likely owing to the presence of unique genes in the exceedingly special species of *C. migao*.

**Fig. 5:**
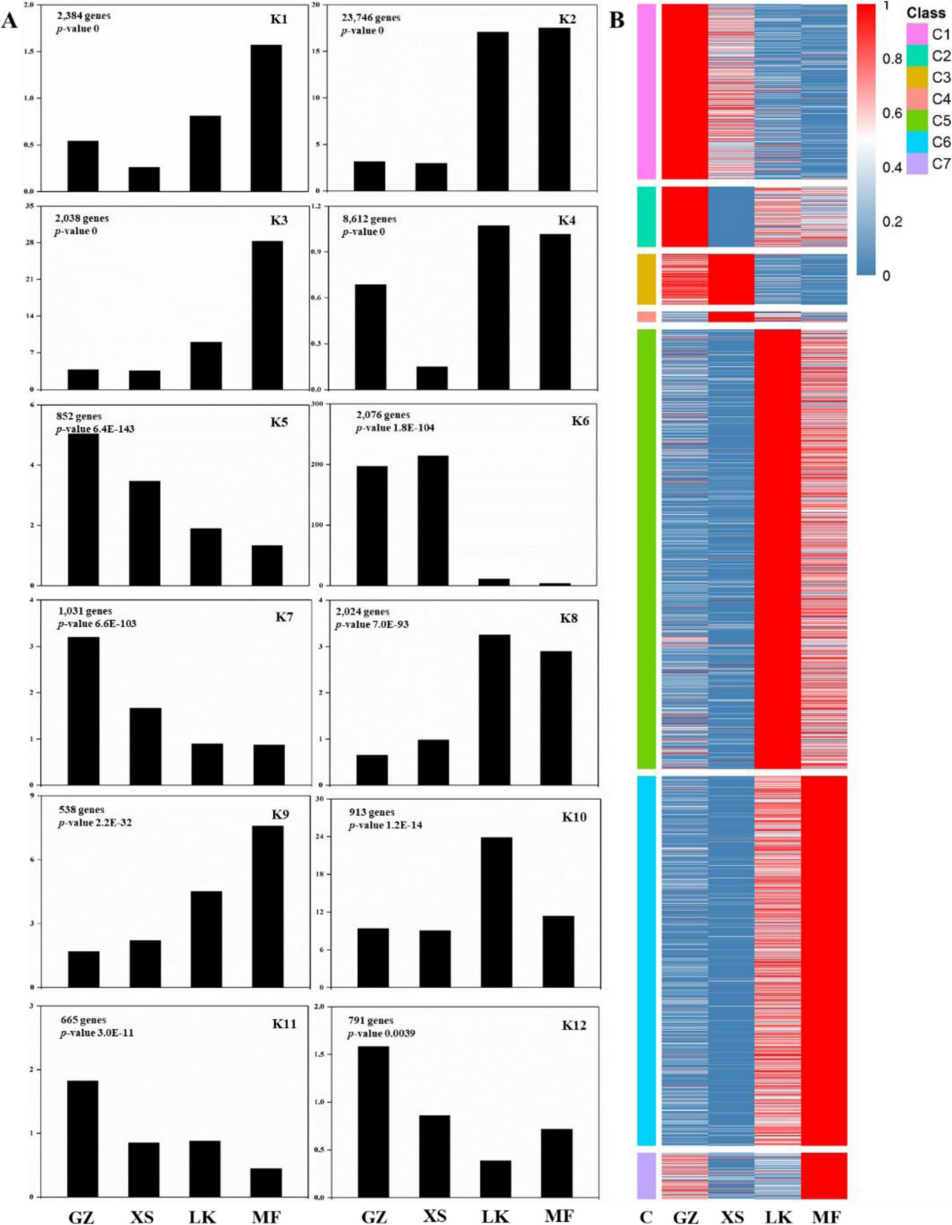
Coordinated changes in the functional gene categories activated during the four stages of seed germination (GZ/XS/LK/MF). **A**. Transcriptional dynamic expression pattern of seed germination. The expression patterns of genes in continuous samples of seed germination were clustered, and the gene sets with certain biological characteristics were selected from the clustering results (*p*-value < 0.05 of each profile). **B**. Cluster analysis of the functional unigenes of seed germination

To obtain the transcriptional dynamic expression pattern during seed germination, we used the STEM software to classify the differentially expressed genes (DEGs) in the different germination stages (GZ, SX, LK, and MF). Meanwhile, a total of 26 expression profiles were obtained, of which 12 different expression patterns (K1–K12, *P* < 0.01) were highly significant (Fig. 5 A). Using hierarchical clustering, we classified the DEGs into seven coexpression classes (C1–C7), each of which contains genes with highly similar expression patterns (Fig. 5B). The expression patterns of the 12 expression profiles were consistent with those of the 7 coexpression classes. The gene expression levels of C1 and C2 were high in the GZ stage, including the genes related to glycosome protein composition, oxidative phosphorylation, and RNA transport. In C3 and C4, expression levels of a small number of genes were upregulated in the XS stage, whereas those of a large number of genes in C5 were continuously upregulated in the LK and MF stages. In C6 and C7, the expression levels of the genes involved in substance and energy metabolism were upregulated in the LK and MF stages and reached the peak in the MF stage; these results were consistent with the physiological changes during seed germination (Fig. 3).

### DEGs and gene ontology and KEGG enrichment

**Fig. 6:**
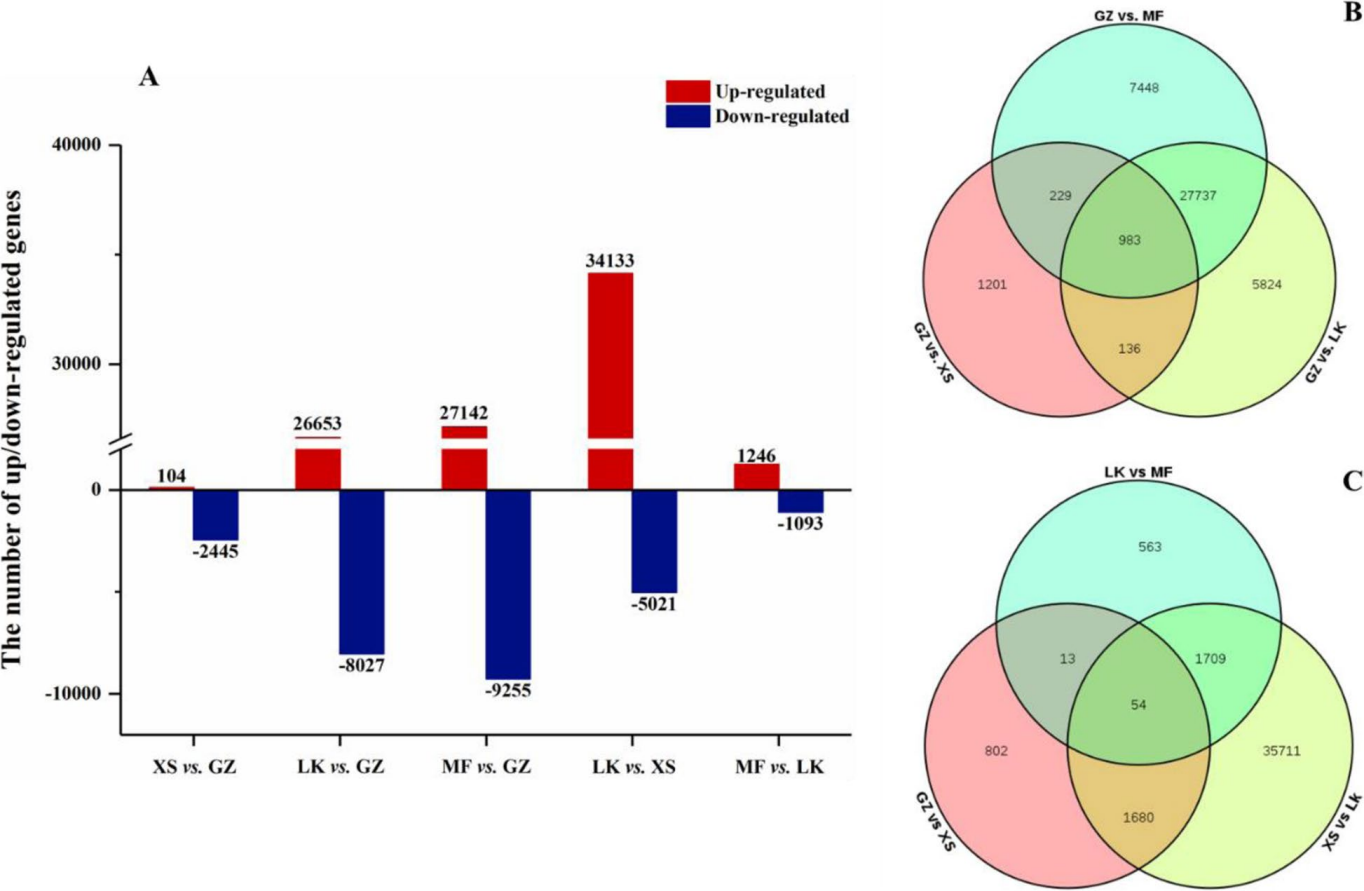
Statistical analysis of the differentially expressed genes in the different stages of *Cinnamomum migao* seed germination. **A**. The number of up/down-regulated genes during seed germination. **B**. Shared DEGs in fixed reference system (FFS) of seed germination. **C**. Shared DEGs in continuous comparison system (CCS) of seed germination

We conducted differential expression analysis of the transcription levels of unigenes in four samples to identify potential regulatory genes involved in seed germination. The DEGs were compared in two main ways: (1) using GZ as the reference point, i.e., the fixed reference system (FFS), and (2) by selecting each previous adjacent time point in turn as the reference point, i.e., the continuous comparison system (CCS). In the FFS and CCS groups of *C. migao* seeds, 43,558 and 40,532 DEGs, respectively, were obtained (Fig. 6 A). The transcript levels of 26,653 genes significantly increased, whereas those of 8,027 genes decreased in the LK stage (LK versus GZ); in contrast, the transcript levels of 34,133 genes markedly increased, whereas those of 5,021 genes decreased in the LK stage (LK versus XS) (Fig. 6 A). In the two comparison systems (FFS and CCS), the DEGs of the MF versus GZ and LK versus XS stages accounted for 83.6 and 96.6 % of the total DEGs of the two groups, respectively; among these, we identified 983 and 54 shared DEGs, respectively (Fig. 6B and C). The results showed that the tissue difference between the seeds in the GZ and MF stages was the largest, whereas in the whole germination process, most DEGs start from the XS stage; the difference was most significant from the XS to LK stage. Analysis of the transcriptional expression levels of the genes in seed germination showed that extensive gene expression occurred during germination.

**Fig. 7:**
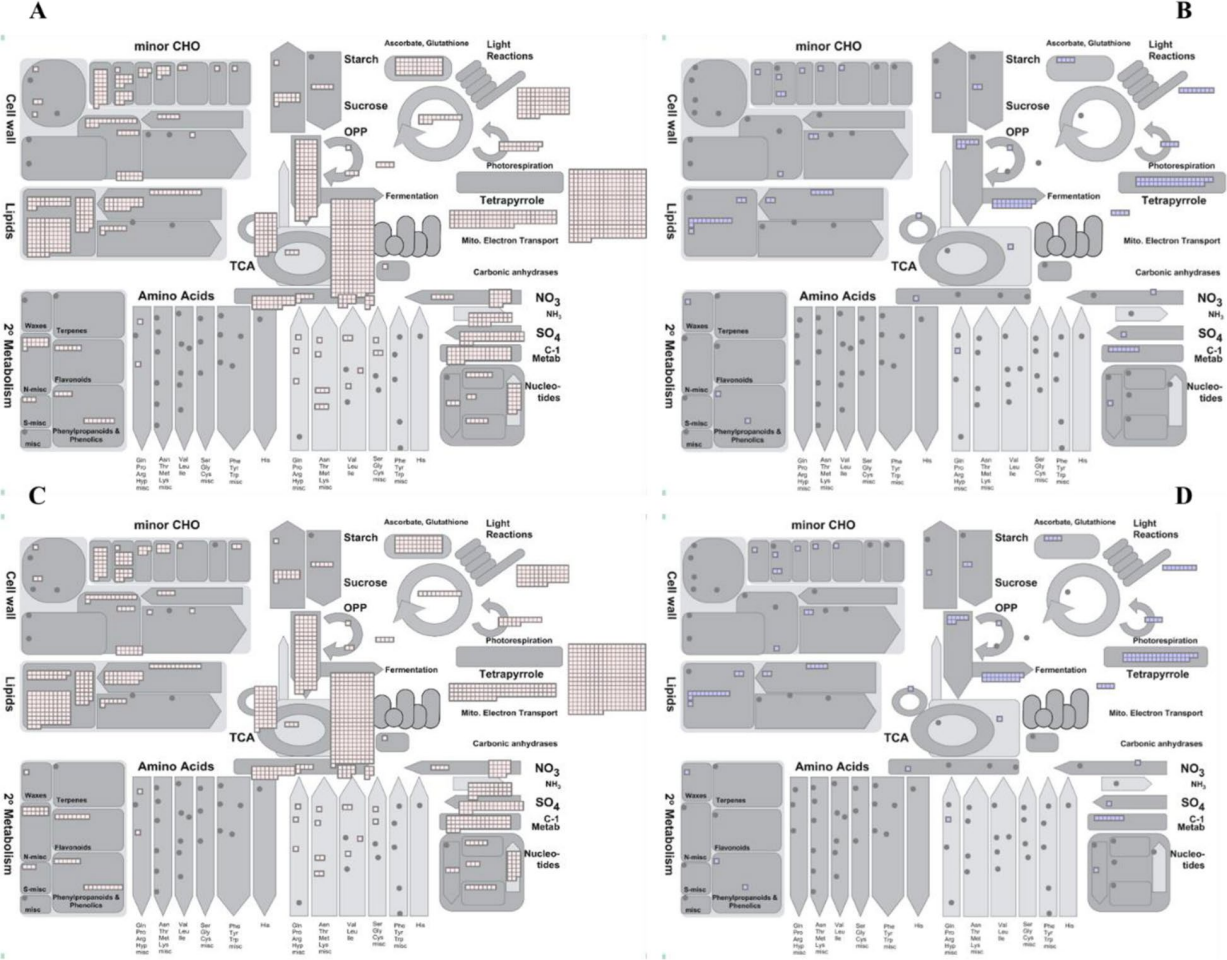
Mapman annotated maps of the differentially expressed genes in the fixed reference system (FFS)and continuous comparison system (CCS) of *Cinnamomum migao* seeds. **A**. MapMan metabolism overview map of the upregulated genes in the FFS group. **B**. MapMan metabolism overview map of the downregulated genes in the FFS group. C. MapMan metabolism overview map of the upregulated genes in the CCS group. **D**. MapMan metabolism overview map of the downregulated genes in the CCS group

In the CCS group of seed germination, consistent or opposite transcript regulation characteristics were observed. In the FFS group, 4,352 MapMan BINs of the upregulated DEGs and 578 MapMan BINs of the downregulated DEGs were obtained; the upregulated genes accounted for 88.28 % of all DEGs. Most DEGs fell into 27 BINs, including RNA, transport, lipid metabolism, protein, carbohydrate, amino acid metabolism, cell, and cell wall (Fig. 7). The upregulated genes were mainly involved in RNA/protein biosynthesis, lipid metabolism, cell respiration, and carbohydrate and nutrient absorption (Fig. 7 A); the downregulated genes were mainly involved in RNA biosynthesis, protein biosynthesis, and solute transport (Fig. 7B). In the CCS group, 4,296 MapMan BINs of the upregulated DEGs and 481 MapMan BINs of the downregulated DEGs were obtained; meanwhile, the classification results of DEGs in the CCS system were similar to those of the FFS group (Fig. 7 C and D).

Because the functional classification generated by MapMan is limited to genes that have putative *Arabidopsis* homologs that have been functionally characterized, we have also utilized GO and KEGG annotations to provide further evidence of *C. migao* functional specialization. Because of the similarity between FFS and CCS, the enrichment analysis of GO and KEGG in DEGs was mainly performed in the CCS group. For GO enrichment, the DEGs in the CCS of XS versus GZ, LK versus XS, and LK versus MF stages were significantly enriched into 48, 81, and 137 GO terms, respectively, which belong to three major functional categories: cell component (C.C.), molecular function (M.F.), and biological process (B.P.). In the LK versus XS stages, the functional categories of the upregulated genes in the LK stage were remarkably enriched in 22 B.P. groups, including cellular process (GO:0009987) and biological regulation (GO:0065007); significantly concentrated in 16 C.C. groups, including cell (GO:0005623), cell part (GO:0044464), and organelle (GO:0043226); and mainly enriched in 11 M.F. groups, including catalytic activity (GO:0003824) and transporter activity (GO:0005215) (Figure S1).

For KEGG enrichment, the DEGs in XS versus GZ, LK versus XS, and LK versus MF stages were significantly enriched into 86, 132, and 114 metabolic pathways, respectively. The upregulated genes in the XS stage (XS versus GZ) were concentrated in protein processing in the endoplasmic reticulum and in cysteine and methionine metabolism (Figure S2A); the downregulated genes in the XS stage were enriched in the ribosome, fatty acid elongation, and plant pathogen interaction (Figure S2B). The upregulated genes in the LK stage (LK versus XS) were mainly concentrated in glycerol metabolism, biosynthesis of amino acids, pentose phosphate pathway (PPP), and TCA (Figure S2C); the downregulated genes in the LK stage were concentrated in endoplasmic reticulum protein processing, ABC transporter, and plant hormones (Figure S2D). The upregulated genes in the MF stage (MF versus LK) were mainly enriched in glyceride metabolism, starch and sucrose metabolism, and glycolysis/gluconeogenesis (Figure S2E); the downregulated genes in the MF stage were concentrated in fatty acid biosynthesis and secondary metabolite biosynthesis (Figure S2F). These results show that several categories have certain characteristics in different stages of germination. Our data provide a unique global view of the gene expression related to material and energy metabolism in *C. migao* seed germination.

### Triacylglycerol (TAG) metabolism in seed germination

The oil content varied with seed germination, and the highest oil content was found in dry seeds (Fig. 3D). The KEGG annotations indicated that 97 DEGs were annotated to glycerolipid metabolism (ko00561), and 160 DEGs were annotated to fatty acid metabolism during seed germination; among them (Figure S3A and S3B), 63 were annotated to fatty acid degradation (ko00071) (Figure S3C) in XS versus LK.

**Fig. 8:**
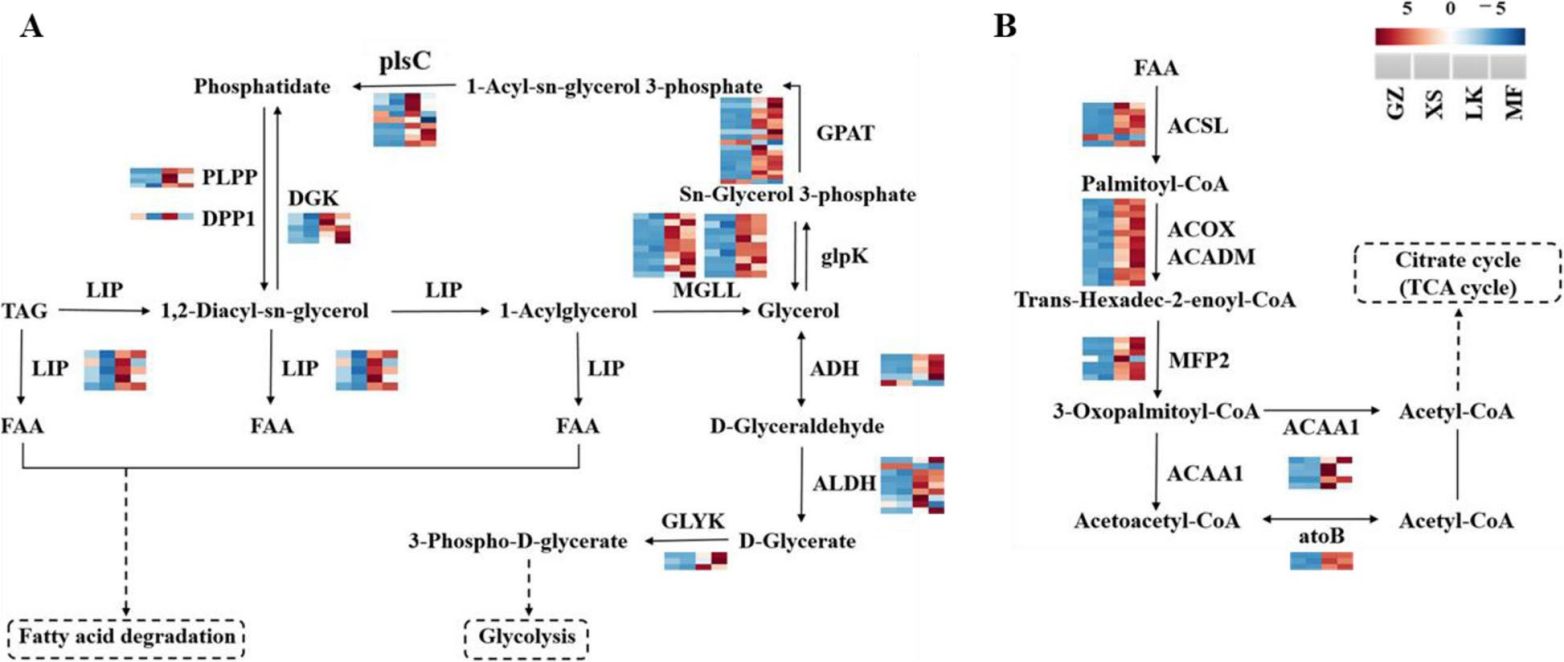
Differentially expressed genes related to the lipid metabolism pathway in the four germination stages of *Cinnamomum migao* seeds. **A**. Glycerol metabolism pathway. **B**. Fatty acid degradation pathway

The DEGs that were annotated to triacylglycerol (oils) decomposition, unigenes encoding triacylglycerol lipase (LIP), acylglycerol lipase (MGLL), glycerol-3-phosphate O-acyltransferase (GPAT), 1-acyl-sn-glycerol-3-phosphate acyltransferase (plsC), phosphatidate phosphatase, phosphatidate phosphatase, alcohol dehydrogenase (ADH), and aldehyde dehydrogenase (ALDH) were more highly expressed in LK and MF seeds (Figure S4). Moreover, in DEGs that underwent fatty acid degradation, the unigenes encoding long-chain acyl-CoA synthetase (ACSL), ACOX, ACADM, MPF2, ACAA1, and atoB were obviously upregulated in LK and MF seeds compared with those in GZ and XS seeds (Fig. 8 A). Hence, the expressions of *MGLL*, *ACSL*, and *ACOX* were upregulated by ≥ 10-fold.

### Starch and sucrose metabolism in seed germination

During the germination process, the starch content of *C. migao* showed a downward trend, whereas that of SSC showed the inverse (Fig. 3). Of the 135 DEGs that were annotated to starch and sucrose metabolism (ko00500), most genes were differentially expressed in the LK versus XS stages, and these DEGs maintained higher expression levels during the LK and MF stages (Figure S4).

**Fig. 9:**
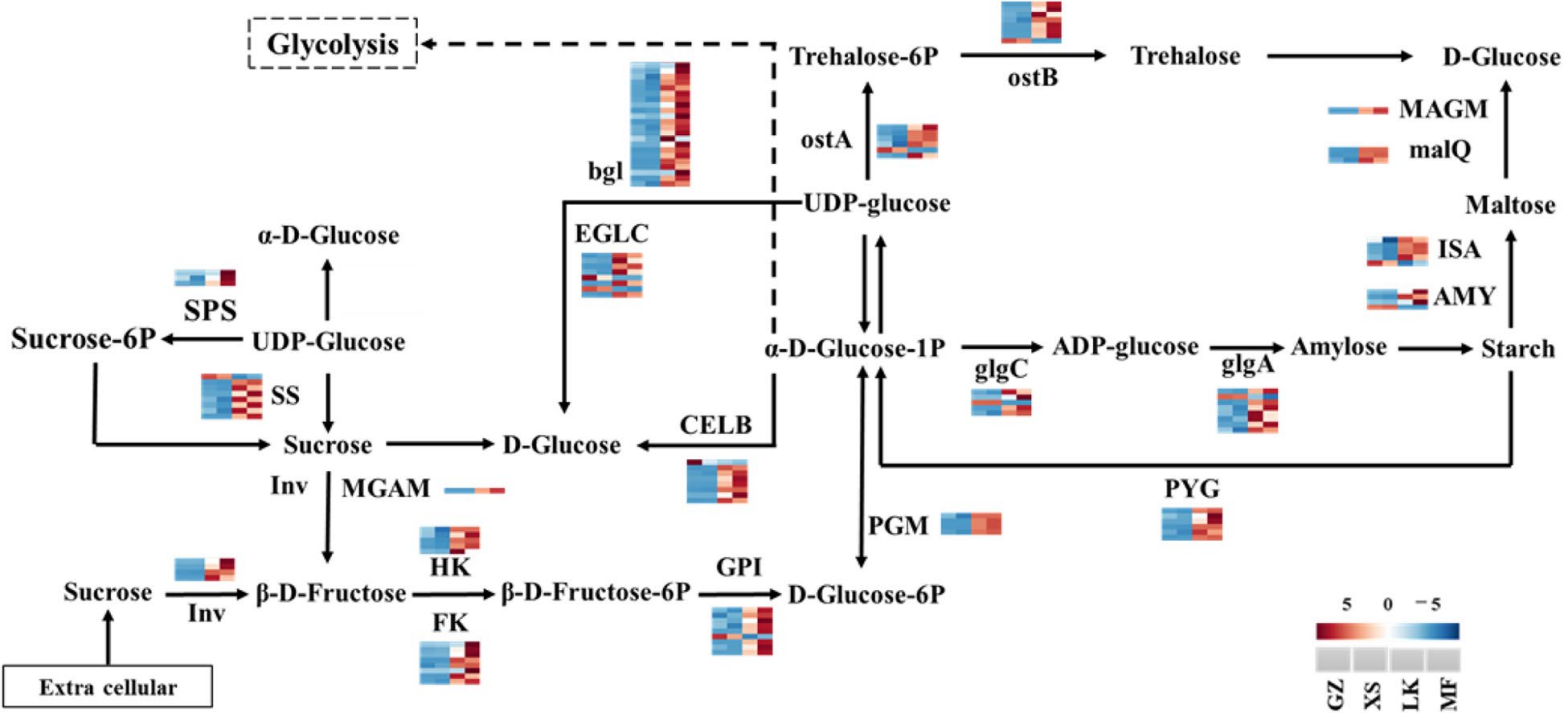
Differentially expressed genes related to the starch and sucrose metabolism pathways in the four germination stages of *Cinnamomum migao* seeds

The expressions levels of unigenes encoding isoamylase (ISA), α-amylase (AMY), maltase-glucoamylase, 4-α-glucosyltransferase, invertase (Inv), hexokinase (HK), fructokinase (FK), glucose-6-phosphate isomerase (GPI), glycogen phosphorylase (PYG), and phosphoglucomutase (PGM) were significantly upregulated during the LK and MF stages, and their upregulated expressions might accelerate the decomposition of starch, maltose, and sucrose in the LK and MF stages. There were still some key genes in starch and sucrose metabolism that were upregulated in the LK and MF stages, including unigenes encoding sucrose synthase (SS), sucrose phosphate synthase (SPS), endoglucanase, β-glucosidase, glucanendo-1,3-β-D-glucosidase endoglucanase, glucose-1-phosphate adenylate transferase (glgC), starch synthase, trehalose 6-phosphatesynthase, and trehalose 6-phosphatephosphatase. Among them, the expression levels of *ISA*, *GPI*, *SS*, and *FK* increased 7- to 16-fold, and the expression levels of these genes were higher in the MF stage than in the LK stage (Fig. 9).

### Energy supply during the germination of seeds

**Fig. 10:**
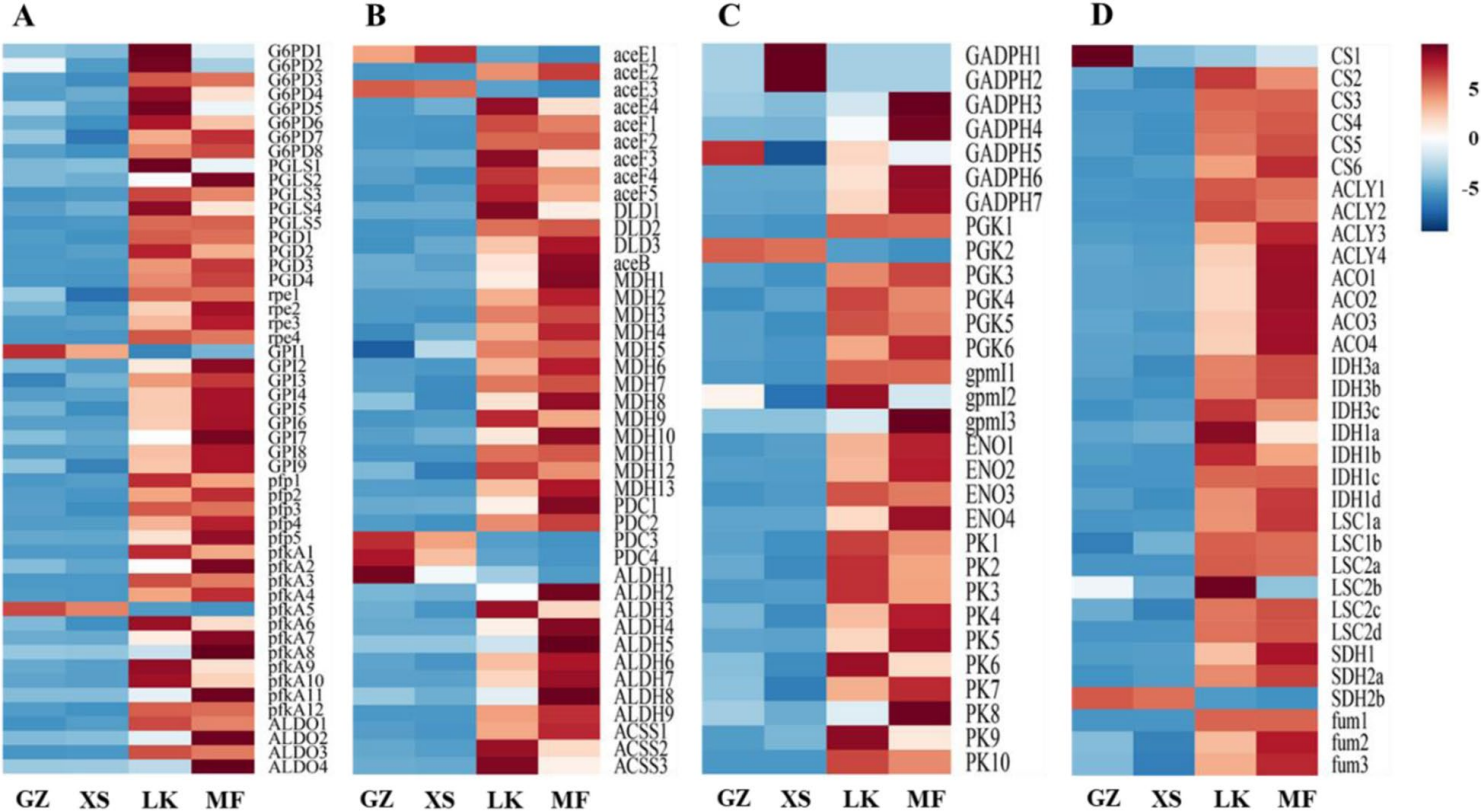
Cluster analysis of the differentially expressed genes (DEGs) related to energy supply in the four germination stages of *Cinnamomum migao* seeds. **A**. Clustering of DEGs related to the pentose phosphate pathway (PPP). **B**. Clustering of DEGs related to glycolysis pathway (EMP). **C**. Clustering of DEGs related to the pyruvate pathway. **D**. Clustering of DEGs related to the tricarboxylic acid (TCA) cycle pathway

The lipids, starches, and sugars in the seeds gradually decomposed via the germination of seeds and then passed through the PPP (ko00030), glycolysis pathway (EMP) (ko00010), pyruvate metabolism (ko00620), TCA cycle (ko00020), and oxidative phosphorylation (ko00190), although they were not completely decomposed. The decomposed products of the macromolecular substances in seeds provide energy for seed germination by changing the expression levels of key genes in these metabolic pathways (Fig. 10 A–D).

**Fig. 11:**
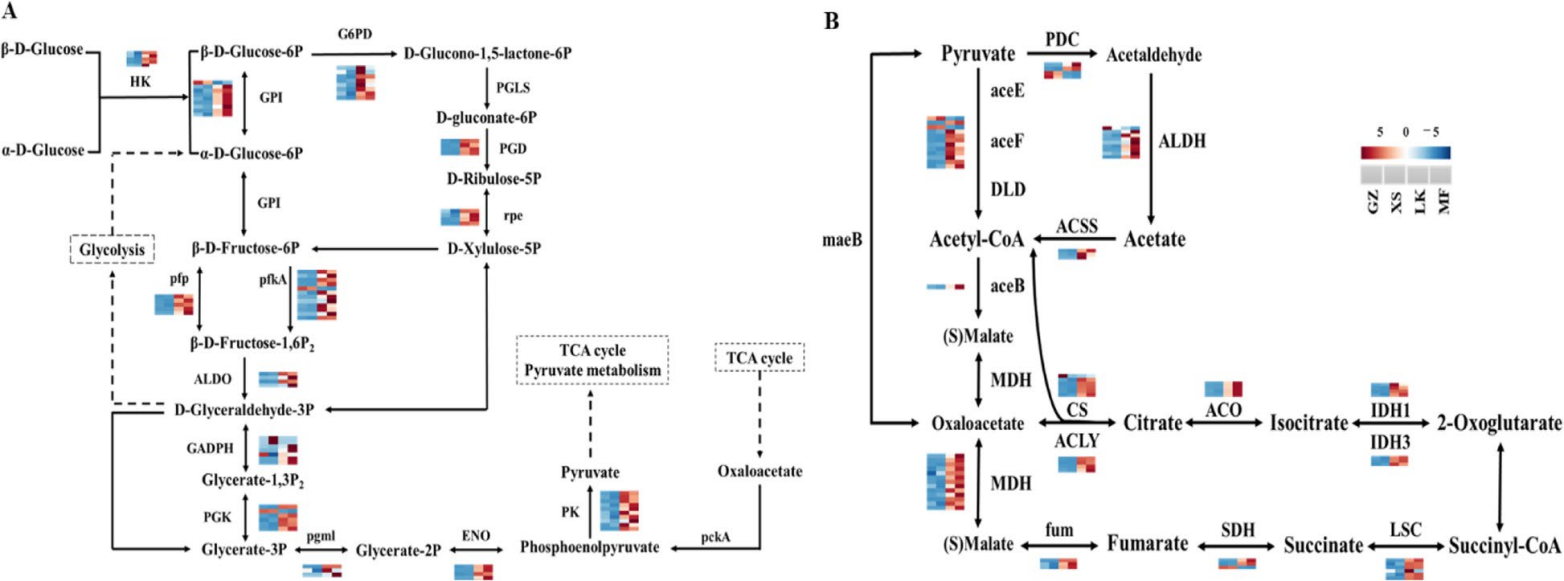
Differentially expressed genes related to energy supply in *Cinnamomum migao* seeds*. ***A**. DEGs in the pathway of PPP and EMP metabolism. **B**. DEGs in the pyruvate pathway and TCA cycle

Glucose undergoes decomposition into D-glyceraldehyde-3P and β-D-fructose-6P in EMP. Of the 49 DEGs annotated to PPP, the unigenes encoding glucose-6-phosphate dehydrogenase (G6PD), phosphor gluconolactonase (PGLS), 6-phosphogluconate dehydrogenase (PGD), ribulose phosphate 3-epimerase, GPI, diphosphate dependent phosphor fructokinase (pfp), 6-phosphofructokinase 1 (pfkA), and fructose bisphosphate aldolase (ALDO) were significantly upregulated in the LK and MF stages (Fig. 10 A). The expressions of unigenes *G6PD*, *PGLS* and *pfp* in the LK stage were 8- to 10-fold higher than those in the XS stage (Fig. 11 A). The changes in the expression of the above genes might accelerate the decomposition of glucose in the seeds in the LK stage. Of the 32 DEGs annotated to EMP, the unigenes encoding glyceraldehyde 3-phosphate dehydrogenase (GADPH), phosphoglycerate kinase (PGK), phosphoglycerate mutase (pgmI), enolase, and pyruvate kinase (PK) were upregulated in the LK stage, and the expressions of *GADPH*, *pgmI, and PK* increased by 9- to 14-fold, which was conducive to the decomposition of D-glyceraldehyde-3P (Fig. 11 A).

Among the 37 DEGs that were annotated to the pyruvate metabolic pathway, the unigenes encoding pyruvate dehydrogenase E1, E2 (aceE, aceF), dihydrolipoamide dehydrogenase (DLD), malate synthase, malate dehydrogenase (MDH), pyruvate decarboxylase (PDC), acetaldehyde dehydrogenase (ALDH), and acetyl-CoA synthetase (ACSS) were upregulated in the LK stage, and the expressions of *MDH*, *maeB*, and *ALDH* increased by 8- to 13-fold (Fig. 10 C). Of the 26 DEGs that were annotated to the TCA cycle, unigenes encoding citrate synthase (CS), ATP citrate (pro-S)-lyase (ACLY), aconitate hydratase (ACO), isocitrate dehydrogenase (IDH), succinyl-CoA synthetase (LSC), succinate dehydrogenase (SDH), and fumarate hydratase (fum) were upregulated in the LK stage (Fig. 10D). In addition, aceE/F, CS, and IDH were the rate-limiting enzymes throughout this process. The expression levels of *ACLY*, *ACO*, *CS*, *IDH*, *LSC*, and *fum* in the LK and MF stages increased by 6- to 10-fold, which might increase the synthesis and decomposition of metabolites in each link, increased the speed of cyclic reaction, accelerated the adjustment of the ATP and NADH concentrations in the LK stage, and ensured the supply of energy required for *C. migao* seed germination (Fig. 11B).

**Fig. 12:**
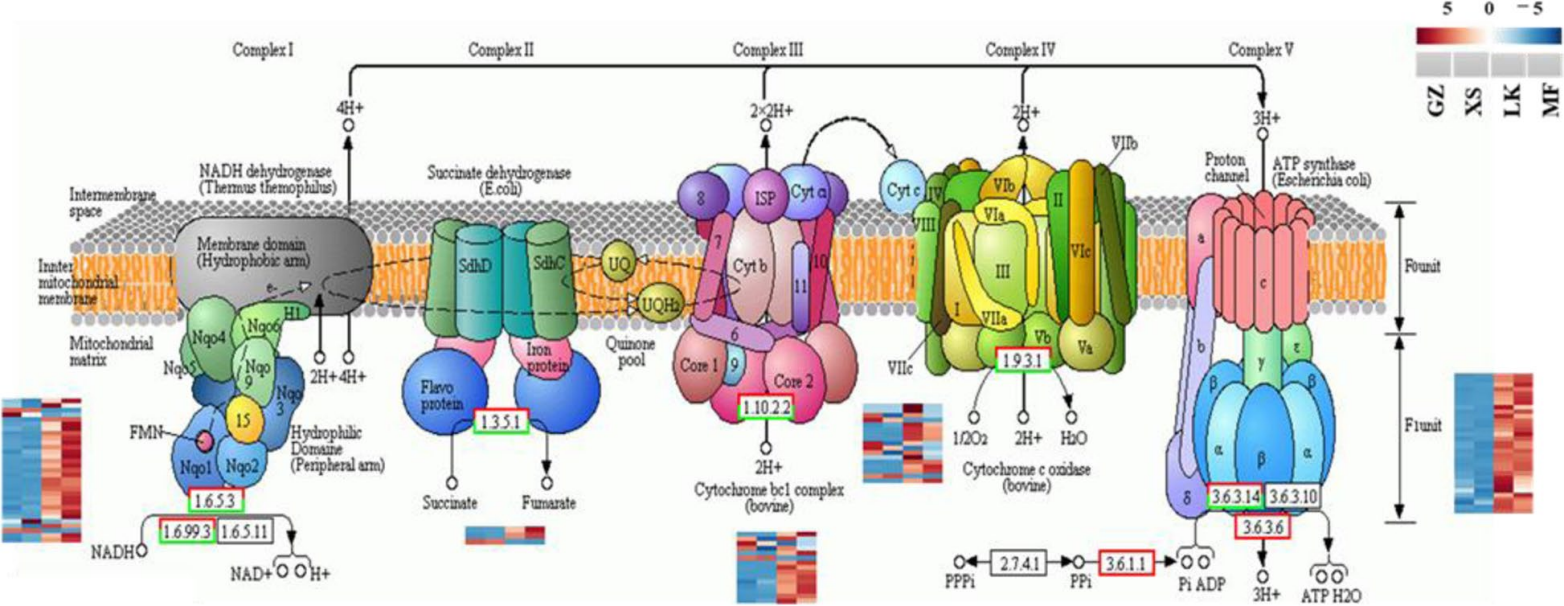
DEGs in the pathway of oxidative phosphorylation pathway in the four germination stages of *Cinnamomum migao* seeds

Of the 92 DEGs that were annotated to oxidative phosphorylation (Figure S5), the unigenes encoding NADH dehydrogenase (NDUF, NDHA, NDHB, and NDHF), succinate deaminase, cytochrome c reductase, cytochrome c oxidase, and transporting ATPase were upregulated in the LK stage, which might accelerate the electron, H^+^ transfer, and oxygen utilization efficiency so that the seeds would generate adequate energy for seed germination in the LK and MF stages (Fig. 12).

### Antioxidant capacity of *C. migao* seeds during germination

**Fig. 13:**
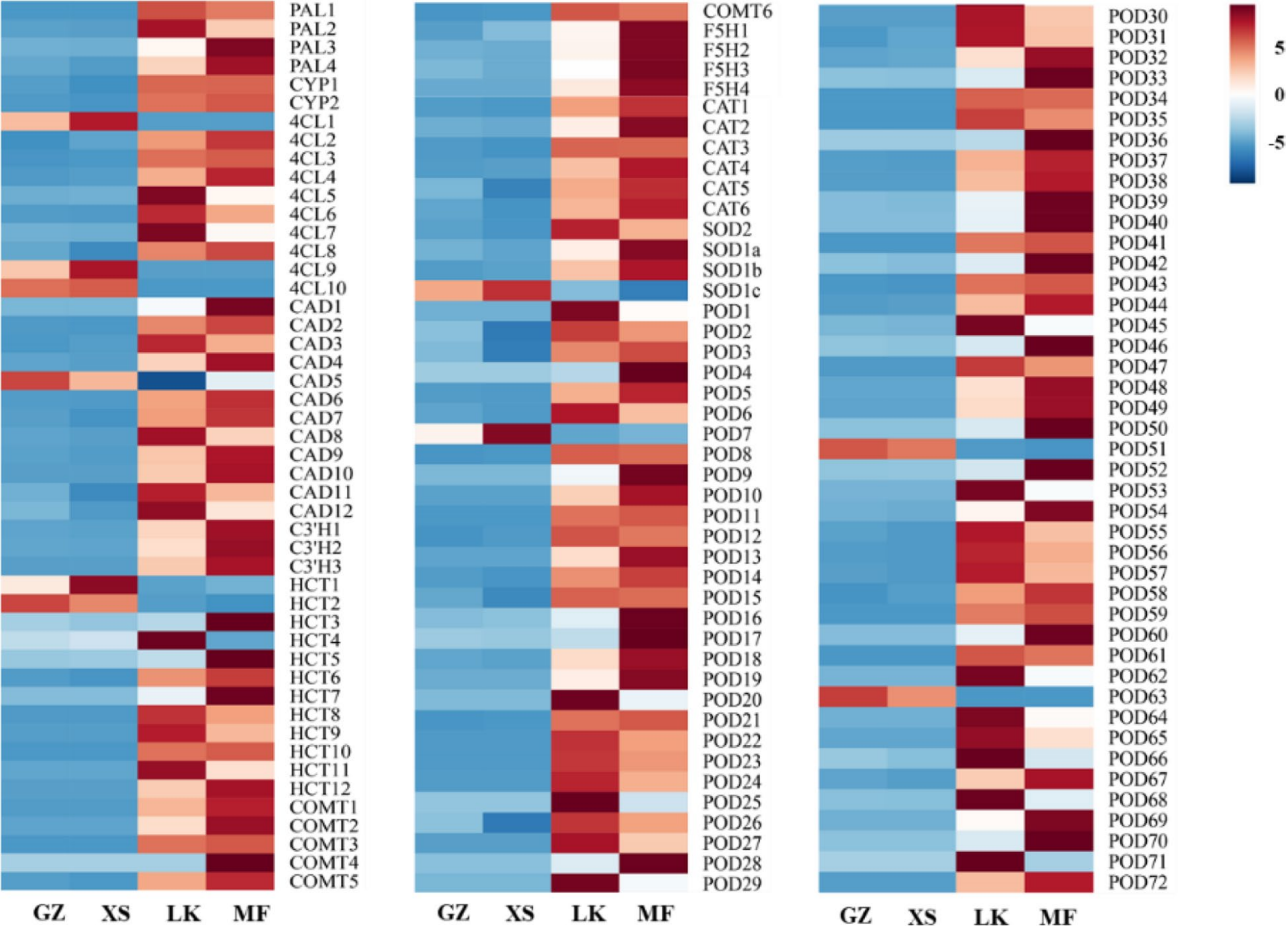
Clustering of the differentially expressed genes related to the antioxidant pathways in the four germination stages of *Cinnamomum migao* seeds

Seeds could activate their defense mechanism in response to biotic and abiotic stress during germination. The annotation of DEGs indicated that the enzymes (SOD, CAT, and POD) scavenging macromolecular reactive oxygen species (ROS) would mainly synthesized by peroxisome (ko04146) and phenylpropanoid biosynthesis (ko00940). During seed germination, nine unigenes encoding CAT and SOD were upregulated in the LK stage. The unigenes of 111 DEGs that were annotated to phenylpropanoid biosynthesis (Fig. 13) encoded phenylalanine ammonia-lyase (PAL), trans-cinnamate 4-monooxygenase (CYP), 4-coumarate-CoA ligase (4CL), cinnamyl-alcohol dehydrogenase, caffeic acid 3-O-methyltransferase (COMT), ferulate-5-hydroxylase (F5H), and POD. It should be noted that the expression of *POD* increased by 17-fold and that of other genes increased by 6–13.8 times during the LK stage; however, the expressions of *PAL*, *HCT*, *CoMT* and *POD* were still upregulated during the MF stage and those of 12 unigenes encoding POD were upregulated and increased by 6.2-fold, which was consistent with the change in the antioxidant enzyme activity during the germination process (Fig. 3 F).

### Metabolic pathway network analysis

**Fig. 14:**
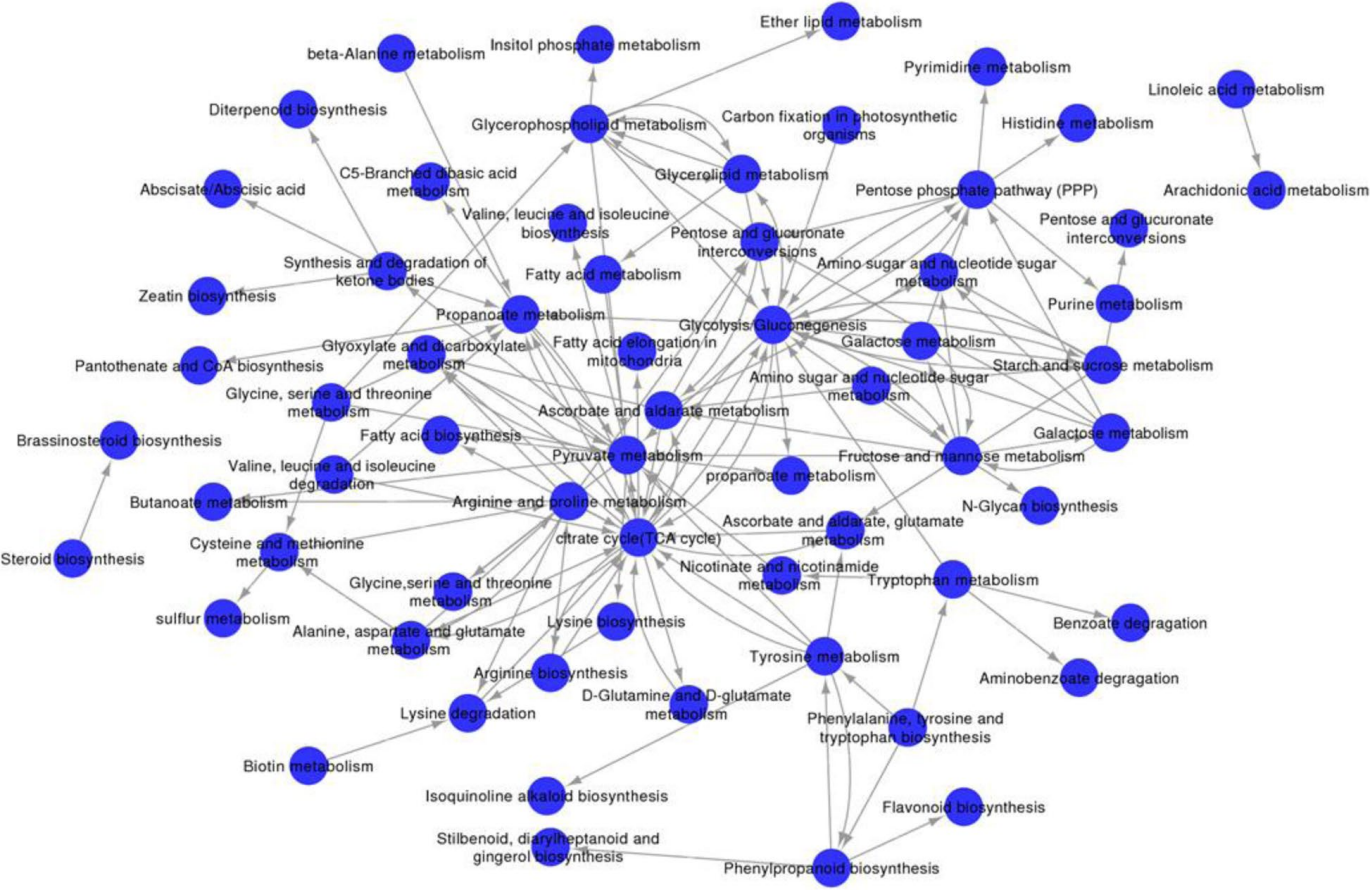
Pathway network of the important pathways involved in *Cinnamomum migao* seed germination*.* Cycle nodes represent different pathways. Arrows represent the interaction between one pathway and another pathway based on their degree. The source and point of the arrow show the source of the pathway and the target of the pathway in the network. The nodes with more arrows indicate that more pathways interact with this node, which shows that the pathway is the most important pathway in the pathway network

According to the interaction between the KEGG enrichment pathways of DEGs in the seeds of *C. migao* (Table S2), a metabolic pathway network was established, which elucidated the interaction among important pathways and determined the activation state among pathways (Fig. 14). Based on the degree of the seeds, the energy metabolism-related pathways, TCA, glycolysis/gluconeogenesis, and pyruvate metabolism were determined to be the center of the network (Fig. 14). The highest degree in the network was attributed to TAC, and the expression levels of 31 unigenes during the TCA cycle were upregulated during the LK stage, which played a key role in providing seed germination energy. The degree of glycolysis/gluconeogenesis was the second highest, and the expressions of 22 unigenes were upregulated during the LK stage. Pyruvate played a connecting role as an important intermediate product in the metabolic network; therefore, the pyruvate metabolic pathway was critical for seed germination, which is involved in lipid biosynthesis, the TCA cycle, and other target metabolic pathways by converting pyruvate to acetyl-CoA and oxaloacetate; meanwhile, 38 unigenes were upregulated during the LK stage (Fig. 11 A). Simultaneously, 49 unigenes in the PPP were upregulated during the LK and MF stages (Fig. 11B), which might promote the decomposition of glucose into D-glyceraldehyde-3P and β-D-fructose-6P in the glycolysis pathway. The metabolism of storage materials (glycerolipid, sucrose, starch metabolism, etc.); amino acid metabolism (tyrosine metabolism, arginine and proline metabolism, lysine degradation, etc.); and intermediates and energy supply pathways (glycolysis, the TCA cycle, PPP, etc.) were systematically triggered in *C. migao* seed germination.

## Discussion

### Metabolism and regulation of storage substances during seed germination

The amount of energy required to support the complex physiological process of seeds germination and the metabolism of storage substances is the key to activate seed germination [24]. Carbohydrates, proteins, and lipids are the main storage substances of most seeds [25], providing the basis for seed germination and playing pivotal roles in seed germination [26]. It has been proven that storage substances with the highest content were mobilized and most used during seed germination. For example, the content of sugar gradually increased during the germination of sorghum seeds, whereas the contents those of the main storage materials fat and starch significantly decreased [27]. The seed germination of *Helianthus annuus* also mainly mobilized and used the oil substances in the seeds, and the oil content sharply decreased during germination [28]. The present study showed that the oil and starch contents significantly decreased during *C. migao* seed germination (Fig. 8) and that there were an abundance of oil bodies (TAG) in the microstructure of embryo cells (Fig. 2); these results indicated that oil and starch are the main storage materials that are mobilized and used. After the seeds entered the XS stage, TAG was mobilized by LIP, and the oil body was gradually and enzymatically hydrolyzed into FAA, which was finally converted into small molecules, such as sugars, serving as energy sources [29]. TAG was decomposed into free FAA and glycerol by lipase; the free FAA was then converted into glyoxysome and then into acetyl-CoA via β-oxidation. Finally, TAG was converted into sugar via the glyoxylic acid cycle and glycolysis or gluconeogenesis [30]. *LIP*, *MGLL*, *GPAT*, *plsC*, *ADH*, and *ALDH* in the glycerolipid metabolism of *C. migao* seeds were upregulated in the LK and MF stages (Fig. 8), thereby accelerating TAG decomposition. Subsequently, *ACSL*, *ACOX*, *MPF2*, *ACAA1*, and other genes in fatty acid degradation were also upregulated in the LK stage, and most of the genes reached the peak at the MF stage (Fig. 8). After a series of reactions, the decomposition products of TAG were transported to the germination and growth regions.

Starch and sucrose play crucial roles in seed germination, and the ultimate purpose of their decomposition is to provide energy for seed germination [31]. Starch is gradually decomposed during the germination of barley seeds, resulting in a decrease in its content [32]; this trend was also observed during the germination of *C. migao* (Fig. 3). Starch hydrolysis depends on ISA, AMY, and β-amylase (BMY). Notably, only one *ISA* and one *BMY* were highly expressed in the GZ and XS stages of *C. migao*, whereas most of the *ISA* and *AMY* were significantly upregulated in the LK and MF stages (Fig. 9). During the germination of *Cyclobalnopsis gilva* seeds, the activity and mRNA expression level of BMY were high; however, no obvious relationship was noted with the activity and expression level of AMY [13]; these results were inconsistent with those of *C. migao* (Fig. 9) and wheat seeds in the XS stage [33]. Another starch decomposition pathway relied on PYG, which decomposed starch into glucose-1-phosphate and then it was transferred to the glycolysis pathway to provide energy for seeds. Notably, the seeds of *C. migao* in the LK and MF stages accelerated starch decomposition by upregulating the expression level of *PYG* (Fig. 9); these results are consistent with those obtained for physiological indicators (Fig. 3). However, wheat seeds with high starch content showed upregulation in the expression level of *PYG* in the XS stage [33]. These results suggest that the starch hydrolysis pathways and amylase species vary among species and that the expression levels of starch hydrolysate genes are also related to the storage substance components of the species.

Soluble sugars, such as sucrose and glucose, which could be used directly during germination [34], are stored in the embryo or endosperm or transformed from oil and starch [35]. During the germination of *Sorghum bicolor* seeds, SSC continuously increased; however, the SSC in the embryos of *C. migao* did not increase until the seeds were completely absorbed by water (Fig. 3). The decrease in SSC per unit weight in the XS stage might be owing to the early absorption of water by the seeds (Fig. 3); these results were consistent with those of seed germination of wild grassland species [27]. Notably, Inv, SPS, SS, GPI, and glgC play key roles in sucrose biosynthesis and metabolism. They convert sucrose into fructose and glucose for glycolysis, thereby providing energy for seed germination [36]. The expression levels of *Inv*, *SS*, *SPS*, and *glgC* remained high during the germination of starchy seeds [37]; however, only *SS*, *GPI*, and *glgC* were upregulated in the GZ and XS stages, whereas the those of other key genes were upregulated in the LK and MF stages (Fig. 9); however, these results were different from those observed in species with high starch content. Owing to the high oil content in *C. migao* seeds, a few unigenes that can decompose starch, sucrose, and oil were upregulated in the GZ and XS stages. The expression levels of TAG and lipid metabolism unigenes began to rapidly increase after entering the LK and MF stages (Fig. 8). In addition, the expression levels of carbohydrate decomposition gene began to be upregulated in the LK stage, but more significantly in the MF stage; the expression levels of TAG and lipid metabolism genes were higher than those of starch metabolism genes (Fig. 8). From the above described physiological, biochemical, and transcriptional results, it is speculated that the seeds of *C. migao* have their own metabolic laws during the prolonged germination cycle.

### Metabolism and regulation of energy during seed germination

The process of seed germination requires enormous amounts of energy to perform physiological activities. Nevertheless, owing to the lack of mineral absorption systems and photosynthetic devices, germinating seeds rely on the mobilization of reserve materials to obtain the necessary energy [34]. Moreover, oxygen content is insufficient in the early stages of seed germination, and glycolysis and alcohol fermentation are the primary energy sources in the early XS stage [33]. Notably, the irreversible enzymes HK, PK, and pfkA are key limiting enzymes in EMP. Therefore, the expression level of the unigenes encoding these enzymes directly determines the speed and direction of the metabolic pathway in the early stage of seed germination. In the GZ and XS stages of *C. migao* seed germination, only one unigene encoding pfkA was upregulated; however, in the LK and MF stages, 26 unigenes encoding HK, PK, and fkA were significantly upregulated (Figs. 9 and 10). In the intermediate stage of *C. migao* seed germination, energy consumption increased and key genes in the glycolysis pathway were upregulated, indicating that the energy requirements significantly increased after XS until the completion of germination; these results were similar to those of maize seed germination [38]. In most grain seeds, energy supply is mainly through the increased expression of *PK* and other genes, which catalyze the production of ATP and pyruvate from phosphoenolpyruvate, which then enters the pyruvate cycle [39]. Moreover, after the XS stage, the expression of key genes involved in glycolysis were upregulated in *C. migao* seeds (Fig. 10). Notably, pyruvate plays the role of an intermediate product and is converted to acetyl-CoA by PDC, ALDH, and ACSS or by ACEE, ACEF, and DLD [40]. The high expression levels of *PDC* and *ALDH* promoted the formation of acetyl-CoA during rice seed germination [33]. Notably, most key genes involved in pyruvate metabolism are upregulated after the XS stage, and acetyl-CoA is mainly produced by the upregulation of *PDC*, *ALDH*, and *ACSS* in the LK stage and by *ace*, *aceF*, and *DLD* in the MF stage (Fig. 10), thereby becoming the energy source for ATP generation in the mitochondrial electron transport chain [31, 41]. Furthermore, PPP is another essential pathway of oxidative decomposition of glucose, and its activation is the key pathway in seed germination, resulting in the generation of D-glyceraldehyde-3P and β-D-fructose-6P, which enter the glycolysis pathway. The expression of key genes involved in PPP was activated during the germination of *Avena fatua* seeds [42]; this result is consistent with that of the current study. The expression levels of *G6PDH*, *PGD*, and *ALDO* in PPP were significantly upregulated (Fig. 11) and peaked in the LK stage (Fig. 11), providing sufficient intermediate materials and energy for seed germination. When the energy provided via anaerobic respiration is insufficient, the TCA cycle provides energy under aerobic conditions for seed germination [33]. The results of maize seed germination revealed that the genes related to TCA cycle and mitochondrial electron transport were upregulated in dry seeds and in the early stage to satisfy the needs of rapid growth after imbibition [38]. *MDH*, *CS*, *ACO*, and *IDH* involved in the TCA cycle were significantly upregulated in the LK and MF stages of *C. migao* seed germination (Fig. 11); this result is similar to that observed in barley seed germination [43]. In addition, ATP generated via electron transfer during oxidative phosphorylation provides energy for seed germination, among which NADH dehydrogenase (complex I) provides long-term high energy requirements, as observed in *Arabidopsis* seed germination and seedling growth [44]. The increase in the mRNA expression level of ATP synthase in the oxidative phosphorylation pathway could promote ATP formation; this was also observed in *Kandelia candel* seed germination [45]. Several unigenes encoding complexes in the oxidative phosphorylation pathway were significantly upregulated in the LK and MF stages (Fig. 12), indicating that this pathway is mainly used to provide energy for *C. migao* seeds in the late germination stage.

### Activation of defense ability during seed germination

Seed germination is related not only to the transformation and usage of storage substances but also to the activation of different responses by various enzymes (antioxidant enzymes) during this process [46]. Enzymatic hydrolysis of proteins provides nitrogen nutrition for seed germination; proteins are components of enzymes and play an important role in seed germination and plants [47]. The soluble protein content increased and reached its peak in the MF stage of *C. migao* seed germination (Fig. 3); this not only increased the activation of the internal physiological activities of the seeds but also maintained the synthesis and metabolism of protective enzymes. This ensured the smooth completion of the cycle from seed dormancy to germination [47]. The respiratory metabolism of seeds increases with the germination process; this results in the production of a large number of ROS, which in turn results in the accumulation of superoxide anion (O^2−^), hydrogen peroxide (H_2_O_2_), and hydroxyl radicals (OH^−^). This ultimately results in lipid peroxidation and oxidative damage of the cell structure as well as an increase in MDA content [48, 49]. During the process of apple seed imbibition and germination, ROS destroy cell lipids in the form of cell messengers or toxic molecules, increase the content of MDA [50], and activate the antioxidant defense system to respond to several biological and abiotic stresses [51].

Previous studies have revealed that SOD disproportionate O_2_^−^ to H_2_O_2_ and O_2_ with less toxicity via the rapid disproportionation reaction [52]. The synergistic effect of CAT and POD could further remove H_2_O_2_, thereby effectively reducing and eliminating the damage caused by free radicals on the cell membrane [53]. During the initial stage of *C. migao* seed germination, the content of MDA increased; moreover, the activity of the protective enzymes SOD, POD, and CAT rapidly increased and remained at relatively high levels (Fig. 3). Although the cell structure was subjected to oxidative stress, the timely clearance of free radicals ensured favorable seed germination. In addition, the increase in the activity of POD can accelerate cell division and new cell wall formation and promote the biosynthesis of lignin, cork layer, and hydroxyproline glycoprotein [54]. Notably, the change in the level of POD activity was the most significant during *C. migao* seed germination, which was similar to the results observed in *Cyclobalanopsis chungii* in seed dormancy release and germination process [55]. Phenylpropanoid metabolism was one of the defense mechanisms [56], in which 111 unigenes were upregulated in the LK and MF stages. Among these unigenes, the expression levels of 68 *POD* genes significantly increased (17-fold) (Fig. 13). The above results indicate that POD play a major role in the ROS scavenging process of the seeds. Nevertheless, POD has been proven to have a positive effect on seed germination [54].

## Conclusions

In this study, RNA-Seq was used to sequence the seeds of *C. migao* for the first time to reveal the gene expression patterns during germination. In addition, the transcription level and sequence of the changes in the transcription levels of *C. migao* seeds during germination were determined. According to the changes, the biological events that occurred at the transcriptional level in the different germination stages could be determined. Lipid metabolism was the main metabolic process that is initially activated and plays significant roles in seed germination. Next, the genes related to starch and sucrose metabolism and energy supply pathways were activated. Simultaneously, the expression of genes related to antioxidant pathways increased, which maintained a suitable germination state. Notably, the unique metabolic sequence of the seed was further confirmed by assessing the cell structure, physiological indicators, and transcriptional expression level, combined with qRT-PCR analysis. These findings provide valuable information regarding the regulation mechanism underlying *C. migao* seed germination. In future studies, we plan to further explore the possible regulatory processes at the transcriptional level.

## Methods

### Plant materials

Fresh mature fruits of the medicinal plant *C. migao* were harvested from Luodian, Guizhou province, Southwest China (25°26′N, 106°31′E) in mid October 2018. The formal identification of the plant material used in this study was performed by professor Jiming Liu of Guizhou university, and the voucher specimen (No. 20,181,015–1, L.D.) of this material has been deposited in the ecological laboratory of Guizhou university. After the skin and flesh were removed, seeds were initially washed 5–6 times with distilled water to remove visible floating particles and then stored in cool and dry ventilated condition for 1 week. Dried seeds were then resterilized with 0.1 % HgCl_2_ for 5 min and rinsed 6–7 times with sterile distilled water (1 min each time). Then, the seeds were incubated at 10 °C/20°C on seed germination. During the germination process, four periods of germination were investigated, namely, stage I (seeds without absorbing water, GZ), stage II (seeds imbibed water for 72 h to full imbibition, XS), stage III (seed coat fissure after imbibition for 24 days, LK), and stage IV [radicle protruding the seed coat (4 mm) after imbibition for 31 days, MF] (Fig. 1). The samples of the four stages were immediately frozen in liquid nitrogen after removing the seed coats and then stored at − 80 °C until further analysis. Three independent biological replicates were performed for all samples, each of which contained 100 seeds for physiological indicators and transcriptome sequencing.

### Seed microstructure observation and determination of physiological indexes

The seeds from the four stages were fixed with 2.5 % glutaraldehyde in phosphate buffer (0.1 M, pH 7.4), dehydrated using a concentration series of ethanol (30 %, 50 %, 70 %, 80 %, 85 %, 90 %, 95 %, 100 %, and 100 % v/v) for 15 min, and then incubated with isoamyl acetate. The samples were treated with vacuum after air drying and were coated with gold in a vacuum evaporator (Quorum). The specimens were viewed and photographed under a HITACHI SU8100 scanning electron microscope. Seeds from the four stages described above were sampled to quantify the physiological parameters; three biological replicates were performed for the four stages. In brief, the soluble sugar content (SSC) and starch in seeds were determined using the anthrone method [57]. Soluble protein content was determined using the Coomassie brilliant blue G250 staining method [58]. A auto fat analyzer was used for *C. migao* lipid determination. MDA content was estimated using the thiobarbituric acid method, as reported by Hodges et al. [59], with minor modifications. SOD activity was analyzed using the nitroblue tetrazolium chloride method described by Lacan and Baccou [60], with minor modifications. POD activity was determined following the guaiacol method [61], with minor modifications. Catalase activity was determined using UV spectrophotometry [62], with minor modifications.

### Transcriptome sequencing and *de novo* assembly

Total RNA was respectively isolated from the seeds of the four germination stages using the Omega Plant RNA kit (No.R6827, Omega Bio-Tek Inc., USA). RNA purity and concentration of all samples were determined using the Nanodrop 2000 spectrophotometer and QUBIT fluorometer (Life Technologies). RNA integrity was determined using the Agilent 2100 bioanalyzer (Agilent Technologies). High-quality RNA with an RNA integrity number of > 8 and of sufficient quantity was used to construct the sequencing library. RNA samples were used for poly(A)^+^selection using oligo (dT) magnetic beads. Next, libraries were sequenced using the Illumina HiSeq™ 4000 platform (Illumina, San Diego, CA, USA) at Gene Denovo Technology Co. Ltd., Guangzhou, China. Raw data of RNA-Seq were collected, and clean data were obtained by removing adapters, unknown nucleotides, and low-quality (Q-value ≤ 10) bases. The Q20, Q30, GC content, and sequence duplication levels of the clean data were simultaneously calculated. High-quality clean data were used for downstream analyses. The clean reads were subsequently assembled *de novo* using the Trinity software (trinityrnaseq-2.0.6, E-value ≤ 0.05) [63].

### Functional annotation and differential expression analysis

To predict the possible functions and biological pathways of the genes, unigene sequences were aligned to the Nr, KOG, Swiss-Prot, GO, and KEGG databases. KEGG pathway mapping for unigenes was performed using the KEGG automatic annotation server [64]. According to the results of Nr annotation, the Blast2GO software (E-value ≤ 0.05) [65] was used to obtain the GO annotation information of unigenes. After obtaining GO annotation, the WEGO software (E-value ≤ 0.05) [66] was used to classify and count the GO functions of all unigenes. Based on the length of the gene and read counts mapped to this gene, the expected number of fragments per kilobase of transcript sequence per million base pairs sequenced of each gene was calculated. The DESeq R package (1.18.0) was used to perform differential expression analysis of the two groups [67]. The false discovery rate (FDR) was used to determine the threshold of the *p*-value in multiple tests [68]. An FDR of < 0.05 and fold change of ≥ 2 were considered the cutoff thresholds to determine the significance of expression.

## Transcriptome expression profiling and functional classification

MapMan was used to determine the gene functional enrichment of seeds germination [69], and the annotated entries with the functional category name “not assigned and unknown” were excluded from the current study. Short Time-series Expression Miner (STEM, http://www.cs.cmu.edu/~jernst/stem) was used to cluster the expression patterns of unigenes in the four germination stages. Maximum Number of Model Profiles: 26. Other parameters: Default values. Function unigenes in the four germination stages were classified into coexpression modules based on the Pearson’s correlation coefficients of the expression profiles among genes using hierarchical clustering [38]. The clusters of significantly regulated genes were assessed using Cluster [70], MeV [71], and TreeView [72].

### Quantitative (q)RT-PCR validation

RNA was extracted from the four germination stages of seeds of three independent biological replications. Twelve mRNA sequences related to starch and lipid metabolism were randomly selected, and genes with different expression patterns were verified via real-time qRT-PCR. Three *C. migao* actin genes were used as reference genes to normalize the expression data. The primer sequences are listed in Table S3. The qRT-PCR verification system used the PowerUp™ SYBR Green Master Mix (ThermoFisher, Chongqing, China) in a volume of 10 µL, which contained 5 µL of SYBR Green Master Mix, 200 ng cDNA template, and 0.4 µM of each of the forward and reverse primers. The qRT-PCR amplification conditions were as follows: 95 °C for 30 s, followed by 40 cycles at 95 °C for 5 s and 60 °C for 30 s (Bio-Rad Laboratories, CA, USA). Relative quantitative data were calculated using the 2^−ΔΔCT^ method [73]. All validations were performed in three biological and technical replicates.

### Statistical analysis

All data were analyzed using the Duncan’s test using the SPSS 21.0 statistical package (Chicago, IL, USA). All data are expressed as mean and standard deviation of at least five replicates. These graphs were built using origin 9.0 (origin lab, Northampton, Ma, USA).

## Supplementary Information

Additional file 1 **Figure S1** GO classification of the differentially expressedgenes in the continuous comparison system of *Cinnamomum migao *seed germination. Theabscissa represents the functional classification and the ordinate representsthe number of genes in the annotation.

Additional file 2 **Figure S2** KEGG enrichment of DEGs in CCS comparison group of *Cinnamomum migao* seed germination.

Additional file 3 **Figure S3** Cluster analysis of differentiallyexpressed genes related to the lipid metabolism pathway in the four germinationstages of *Cinnamomum migao *seeds.A, B. Cluster analysis of differentially expressed genes related to glycerolmetabolism pathway. C. Cluster analysis of differentially expressed genesrelated to fatty acid degradation pathway.

Additional file 4 **Figure S4** Clusteranalysis of differentially expressed genes related tothe starch and sucrose metabolism pathways in the four germination stages of *Cinnamomum migao* seeds.

Additional file 5 **Figure S5** Clustering ofdifferentially expressed genes related to oxidative phosphorylation pathway in the four germination stages of *Cinnamomummigao* seeds.

Additional file 6 **Table S1** Statistics ofdifferent germination stages sequencing data of *Cinnamomum migao* seeds. a. Before filter reads number, b. Afterfilter high quality reads number, c. After filter clean data bases, d. Afterfilter high-quality clean data bases.

Additional file 7 **Table S2** Source andtarget pathways of the pathway-net during *Cinnamomummigao* seed germination. Degree means the number of relationships that onepathway has with other pathways, the value of degree represents theirsignificance. Indegree is the target pathway, outdegree is the source pathway.

Additional file 8 **Table S3** Sequences ofprimers used for qRT-PCR.

## Data Availability

The raw sequence data from this study have been deposited in the publicly accessible NCBI Sequence Read Archive (SRA) database as accession number PRJNA724866. The datasets supporting the conclusions of this article are included within the article and its additional files. The datasets used and/or analyzed during the current study are available from the authors on reasonable request (Xiaolong Huang, guidah365@126.com; Jiming Liu, karst0623@163.com ).

## Abbreviations

Nr: Nonredundant Protein Sequences
COG: Clusters of Orthologous Groups of Proteins
Swiss-Prot: Swiss-prot protein database
GO: Gene Ontology
KEGG: Kyoto Encyclopedia of Genes and Genomics

## References

[1] Zhang X, Zhou T, Guo L, Huang L, Jiang W, Yang Z, Ma C (2011). Volatile oil contents correlate with geographical distribution patterns of the miao ethnic herb fructus *cinnamomi*. Acta Ecol Sinica.

[2] Zhao S, Li HY, Qiu DW, Liu ZR, Liu N (1991). *Cinnamomum migao* resources and ecological investigation: guizhou, northern guangxi and the contiguous areas of hunan, guizhou and guangxi. J Guiyang U tradit Chin Med.

[3] Li L, Liu J, Huang X, Luo C, Xiong X, Liu J, Li J, Deng M (2018). Genetic diversity of *Cinnamomum migao* populations using ISSR markers. J Northwest A F U.

[4] Rajjou L, Duval M, Gallardo K, Catusse J, Bally J, Job C, Job D (2012). Seed germination and vigor. Annu Rev Plant Biol.

[5] Zaynab M, Kanwal S, Furqan M, Islam W, Noman A, Ali GM, Rehman N, Zafar S, Sughra K, Jahanzab M (2017). Proteomic approach to address low seed germination in cyclobalnopsis gilva. Biotechnol Lett.

[6] Bewley JD (1997). Seed germination and dormancy. Plant Cell.

[7] Han C, Yang P. Studies on the molecular mechanisms of seed germination. Proteomics 2015, 15. 10.1002/pmic.201400375. 10.1002/pmic.201400375 25597791

[8] Mangrauthia S, Surekha A, Sailaja B, Neelamraju S, Voleti S. Transcriptome analysis of *Oryza sativa* (rice) seed germination at high temperature shows dynamics of genome expression associated with hormones signalling and abiotic stress pathways. Trop Plant Biol 2016, 9. 10.1007/s12042-016-9170-7.

[9] Ortiz-Espín AM, Iglesias-Fernández R, Calderón A, Carbonero P, Sevilla F, Jiménez A. Mitochondrial AtTrxo1 is transcriptionally regulated by AtbZIP9 and AtAZF2 and affects seed germination under saline conditions. J Exp Bot 2017, 68. 10.1093/jxb/erx012. 10.1093/jxb/erx012 PMC5441863 28184497

[10] Nonogaki H, Bassel G, Bewley J (2010). Germination—still a mystery. Plant Sci.

[11] Nonogaki H (2014). Seed dormancy and germination-emergin mechanisms and new hypothesis. Front Plant Sci.

[12] Rabelo DB, Gamosa E, Ribeiro E, Costa E, Oliveira A, Venancio T (2016). Transcriptome analysis uncovers key regulatory and metabolic aspects of soybean embryonic axes during germination. Sci Rep.

[13] Zaynab M, Pan D, Fatima M, Chen W (2018). Transcriptomic approach to address low germination rate in *Cyclobalnopsis gilva* seeds. S Afr J Bot.

[14] Pergo E, Ishii-Iwamoto E (2011). Changes in energy metabolism and antioxidant defense systems during seed germination of the weed species *Ipomoea triloba* L. and the responses to allelochemicals. J Chem Ecol.

[15] Bao Y, Yao Z, Cao X, Peng J, Xu Y, Chen M, Zhao S (2017). Transcriptome analysis of *Phelipanche aegyptiaca* seed germination mechanisms stimulated by fluridone, tis108, and gr24. Plos One.

[16] Noman A, Ali S, Naheed F, Ali Q, Farid M, Rizwan M, Irshad MK (2015). Foliar application of ascorbate enhances the physiological and biochemical attributes of maize (*Zea mays* L.) cultivars under drought stress. Arch Agron Soil Sci.

[17] Bewley J. Protein and nucleic acid synthesis during seed germination and early seedling growth. 1982: 559–591. 10.1007/978-3-642-68237-7_16.

[18] Rajjou L, Belghazi M, Huguet R, Robin C, Moreau A, Job C, Job D (2006). Proteomic investigation of the effect of salicylic acid on *Arabidopsis* seed germination and establishment of early defense mechanisms. Plant Physiol.

[19] Wang L, Wang H, Yin L, Tian C. Transcriptome assembly in *Suaeda aralocaspica* to reveal the distinct temporal gene/mirna alterations between the dimorphic seeds during germination. BMC Genom. 2017, 18. 10.1186/s12864-017-4209-1. 10.1186/s12864-017-4209-1 PMC5649071 29052505

[20] Liew LC, Narsai R, Wang Y, Berkowitz O, Whelan J, Lewsey MG (2020). Temporal tissue-specific regulation of transcriptomes during barley (*Hordeum vulgare*) seed germination. The Plant Journal.

[21] Zhu L, Zhao X, Xu Y, Wang Q, Wang H, Wu D, Jiang L (2020). Effect of germination potential on storage lipids and transcriptome changes in premature developing seeds of oilseed rape (*Brassica napus* L.). Theor Appl Genet.

[22] Sun J, Wang P, Zhou T, Rong J, Jia H, Liu Z. Transcriptome analysis of the effects of shell removal and exogenous gibberellin on germination of *Zanthoxylum* seeds. Sci Rep 2017, 7. 10.1038/s41598-017-07424-0. 10.1038/s41598-017-07424-0 PMC5561108 28819199

[23] Shu K, Liu X, Xie Q, He Z (2016). Two faces of one seed: hormonal regulation of dormancy and germination. Mol Plant.

[24] Kazmi RH, Willems LAJ, Joosen RVL, Khan N, Ligterink W, Hilhorst HWM (2017). Metabolomic analysis of tomato seed germination. Metabolomics.

[25] Alencar N, Innecco R, Gomes-Filho E, Gallao M, Alvarez-Pizarro J, Prisco JT, de Oliveira A (2012). Seed reserve composition and mobilization during germination and early seedling establishment of *Cereus jamacaru* d.c. Ssp. *Jamacaru* (Cactaceae). An Acad Bras Ciênc.

[26] Yang Q, Sang S, Chen Y, Wei Z, Wang P (2017). The role of *arabidopsis* inositol polyphosphate kinase ATIPK2β in glucose suppression of seed germination and seedling development. Plant Cell Physiol.

[27] Yang R, Wang P, Elbaloula M, Gu Z (2016). Effect of germination on main physiology and biochemistry metabolism of sorghum seeds. Biosci J.

[28] Erbaş S, Şanlı A (2016). Mobilization of seed reserves during germination and early seedling growth of two sunflower cultivars. J Appl Bot Food Qual.

[29] Bewley JD, Bradford K, Hilhorst H, Nonogaki H. Seeds: physiology of development, germination and dormancy, 3rd edition; 2013. 10.1007/978-1-4614-4693-4.

[30] Eastmond P (2006). Sugar-dependent1 encodes a patatin domain triacylglycerol lipase that initiates storage oil breakdown in germinating *Arabidopsis* seeds. The Plant cell.

[31] Weitbrecht K, Mueller K, Leubner G (2011). First off the mark: early seed germination. J Exp Bot.

[32] Quek WP, Yu W, Tao K, Fox GP, Gilbert RG (2019). Starch structure-property relations as a function of barley germination times. Int J Biol Macromol.

[33] Yu Y, Guo G, Lv D, Hu Y, Li J, Yan Y (2014). Transcriptome analysis during seed germination of elite chinese bread wheat cultivar jimai 20. BMC Plant Biol.

[34] Zhao M, Zhang H, Yan H, Qiu L, Baskin C. Mobilization and role of starch, protein, and fat reserves during seed germination of six wild grassland species. Front Plant Sci 2018, 9. 10.3389/fpls.2018.00234. 10.3389/fpls.2018.00234 PMC5835038 29535748

[35] Sharma S, Sakshi G, Munshi S. Changes in lipid and carbohydrate composition of germinating soybean seeds under different storage conditions. Asian J Plant Sci 2007, 6. 10.3923/ajps.2007.502.507.

[36] Miransari M, Smith DL (2014). Plant hormones and seed germination. Environ Exp Bot.

[37] Liu B, Zhang N, Wen Y, Jin X, Yang J, Si H, Wang D (2015). Transcriptomic changes during tuber dormancy release process revealed by RNA sequencing in potato. J Biotechnol.

[38] Liu W, Chang Y, Chen SC, Lu C, Wu Y, Lu MJ, Chen D, Shih AC, Sheue C, Huang H. Anatomical and transcriptional dynamics of maize embryonic leaves during seed germination. Proc Natl Acad Sci Unit States Am. 2013,110(10): 3979–84. 10.1073/pnas.1301009110. 10.1073/pnas.1301009110 PMC3593892 23431200

[39] Yang P, Li X, Wang X, Chen H, Chen F, Shen S (2007). Proteomic analysis of rice (*Oryza sativa*) seeds during germination. Proteomics.

[40] Gass N, Glagotskaia T, Mellema S, Stuurman J, Barone M, Mandel T, Roessner-Tunali U, Kuhlemeier C (2005). Pyruvate decarboxylase provides growing pollen tubes with a competitive advantage in petunia. Plant Cell.

[41] Li J, Lv X, Wang L, Qiu Z, Song X, Lin J, Chen W (2017). Transcriptome analysis reveals the accumulation mechanism of anthocyanins in ‘zijuan’ tea (*Camellia sinensis* var. Asssamica (masters) kitamura) leaves. Plant Growth Regul.

[42] Simmonds J, Simpson G (2011). Increased participation of ppp in response to after-ripening and gibberellic acid treatment in caryopses of *Avena fatua*. Can J Bot.

[43] Sreenivasulu N, Usadel BOR, Winter A, Radchuk V, Scholz U, Stein N, Weschke W, Strickert M, Close TJ, Stitt M (2008). Barley grain maturation and germination: metabolic pathway and regulatory network commonalities and differences highlighted by new Mapman/pageman profiling tools. Plant Physiol.

[44] Rosental L, Nonogaki H, Fait A (2014). Activation and regulation of primary metabolism during seed germination. Seed Sci Res.

[45] Pan D, Wang L, Chen S, Lv X, Lu S, Cheng C, Tan F, Chen W (2018). Protein acetylation as a mechanism for kandelia candel’s adaption to daily flooding. Tree Physiol.

[46] Xu L, Wang P, Ali B, Yang N, Chen Y, Wu F, Xu X (2017). Changes of the phenolic compounds and antioxidant activities in germinated adlay seeds. J Sci Food Agr.

[47] Hao T, Ding XT, Zhang HM, Jin HJ, Yu JZ, Zhu YL (2014). Growth and physiological reaction of different cucurbit crops in the high root-zone temperature stress. Plant Physiol J.

[48] Wojtyla A, Lechowska K, Kubala S, Garnczarska M. Different modes of hydrogen peroxide action during seed germination. Front Plant Sci 2016, 7. 10.3389/fpls.2016.00066. 10.3389/fpls.2016.00066 PMC4740362 26870076

[49] Huang Y, Lin C, He F, Li Z, Guan Y, Hu Q, Hu J (2017). Exogenous spermidine improves seed germination of sweet corn via involvement in phytohormone interactions, H_2_O_2_ and relevant gene expression. BMC Plant Biol.

[50] Ciacka K, Krasuska U, Otulak K, Gniazdowska A. Dormancy removal by cold stratification increases glutathione and s-nitrosoglutathione content in apple seeds. Plant Physiol Bioch. 2019, 138. 10.1016/j.plaphy.2019.02.026. 10.1016/j.plaphy.2019.02.026 30861401

[51] Worarad K, Suzuki T, Ishii K, Kozaki T, Iigo M, Yamane K (2016). Transcriptome analysis of seed dormancy after rinsing and chilling in ornamental peaches (*Prunus persica* (L.) Batsch). BMC Genomics.

[52] Amooaghaie R (2017). Triangular interplay between ROS, ABA and GA in dormancy alleviation of *Bunium persicum* seeds by cold stratification. Russ J Plant Physl.

[53] Marta B, Szafrańska K, Posmyk M. Exogenous melatonin improves antioxidant defense in cucumber seeds (*Cucumis sativus* L.) Germinated under chilling stress. Front Plant Sci 2016, 7. 10.3389/fpls.2016.00575. 10.3389/fpls.2016.00575 PMC4848318 27200048

[54] Zhang W, Fan J, Tan Q, Zhao M, Zhou T, Cao F (2017). The effects of exogenous hormones on rooting process and the activities of key enzymes of *Malus hupehensis* stem cuttings. Plos One.

[55] Huang YR, Zhuang K, Wu PF, Ma XQ, Lai XL, Tang WM (2017). Seed germination and growth characteristics of *Cyclobalanopsis chungii*. Chin J Ecol.

[56] Tang Q, Ma X, Mo C, Wilson I, Song C, Zhao H, Yang Y, Fu W, Qiu D (2011). An efficient approach to finding *Siraitia grosvenorii* triterpene biosynthetic genes by RNA-seq and digital gene expression analysis. BMC Genomics.

[57] Qu J, Shutu X, Tian X, Li T, Wang L, Zhong Y, Xue J, Guo D (2019). Comparative transcriptomics reveals the difference in early endosperm development between maize with different amylose contents. Peerj.

[58] Bradford MM (1976). A rapid and sensitive method for the quantitation of microgram quantities of protein utilizing the principle of protein-dye binding. Anal Biochem.

[59] Hodges DM, DeLong JM, Forney CF, Prange RK (1999). Improving the thiobarbituric acid-reactive-substances assay for estimating lipid peroxidation in plant tissues containing anthocyanin and other interfering compounds. Planta.

[60] Lacan D, Baccou JC. High levels of antioxidant enzymes correlate with delayed senescence in nonnetted mus kmelon fruits. Planta 1998(204):377–82. 10.1007/s004250050269.

[61] Venisse J, Malnoy M, Faize M, Paulin J, Brisset M. Modulation of defense responses of *Malus* spp. during compatible and incompatible interactions with erwinia amylovora. Mol Plant Microbe In. 2002, 15(12): 1204–12. 10.1094/MPMI.2002.15.12.1204. 10.1094/MPMI.2002.15.12.1204 12481992

[62] Cakmak I, Marschner H (1992). Magnesium deficiency and high light intensity enhance activities of superoxide dismutase, ascorbate peroxidase, and glutathione reductase in bean leaves. Plant Physiol.

[63] Grabherr MG, Haas BJ, Yassour M, Levin JZ, Thompson DA, Amit I, Adiconis X, Fan L, Raychowdhury R, Zeng Q (1992). Magnesium deficiency and high light intensity enhance activities of superoxide dismutase, ascorbate peroxidase, and glutathione reductase in bean leaves. Plant Physiol.

[64] Kanehisa M, Goto S (2000). Kegg: kyoto encyclopedia of genes and genomes. Nucleic Acids Res.

[65] Conesa A, Götz S, García-Gómez J, Terol J, Talon M, Robles M (2005). Blast2GO: a universal tool for annotation, visualization and analysis in functional genomics research. Bioinformatics.

[66] Jia Y, Lin F, Zheng HK, Zhang Y, Chen J, Zhang ZJ, Jing W, Li ST, Li RQ, Lars B (2006). Wego: a web tool for plotting GO annotations. Nucleic Acids Res.

[67] Li B, Dewey CN (2011). Rsem: accurate transcript quantification from rna-seq data with or without a reference genome. BMC Bioinformatics.

[68] Benjamini Y, Hochberg Y (1995). Controlling the false discovery rate - a practical and powerful approach to multiple testing. J Royal Statist Soc Series B.

[69] Schwacke R, Ponce-Soto GY, Krause K, Bolger AM, Arsova B, Hallab A, Gruden K, Stitt M, Bolger ME, Usadel B (2019). Mapman4: a refined protein classification and annotation framework applicable to multi-omics data analysis. Mol Plant.

[70] Brown P, Botstein D, Eisen BM, Spellman P (1998). Cluster analysis and display of genome-wide expression patterns. Proc Natl Acad Sci U S A.

[71] Howe EA, Sinha R, Schlauch D (2011). Quackenbush J RNA-seq analysis in mev. Bioinformatics.

[72] Saldanha AJ (2004). Java treeview-extensible visualization of microarray data. Bioinformatics.

[73] Schmittgen T, Livak K (2008). Analyzing real-time PCR data by the comparative *C*_T_ method. Nat Protoc.

