# Supplementary material for: Transcriptome analysis of Cinnamomum migao seed germination in medicinal plants of Southwest China
Source: BMC Plant Biol. 2021 Jun 11;21:270. doi: 10.1186/s12870-021-03020-7 (PMC8194011; doi:10.1186/s12870-021-03020-7)
Supplement: Supplementary file 1 — Figure S1 GO classification of the differentially expressedgenes in the continuous comparison system of Cinnamomum migao seed germination. Theabscissa represents the functional classification and the ordinate representsthe number of genes in the annotation. [file 12870_2021_3020_MOESM1_ESM.docx]

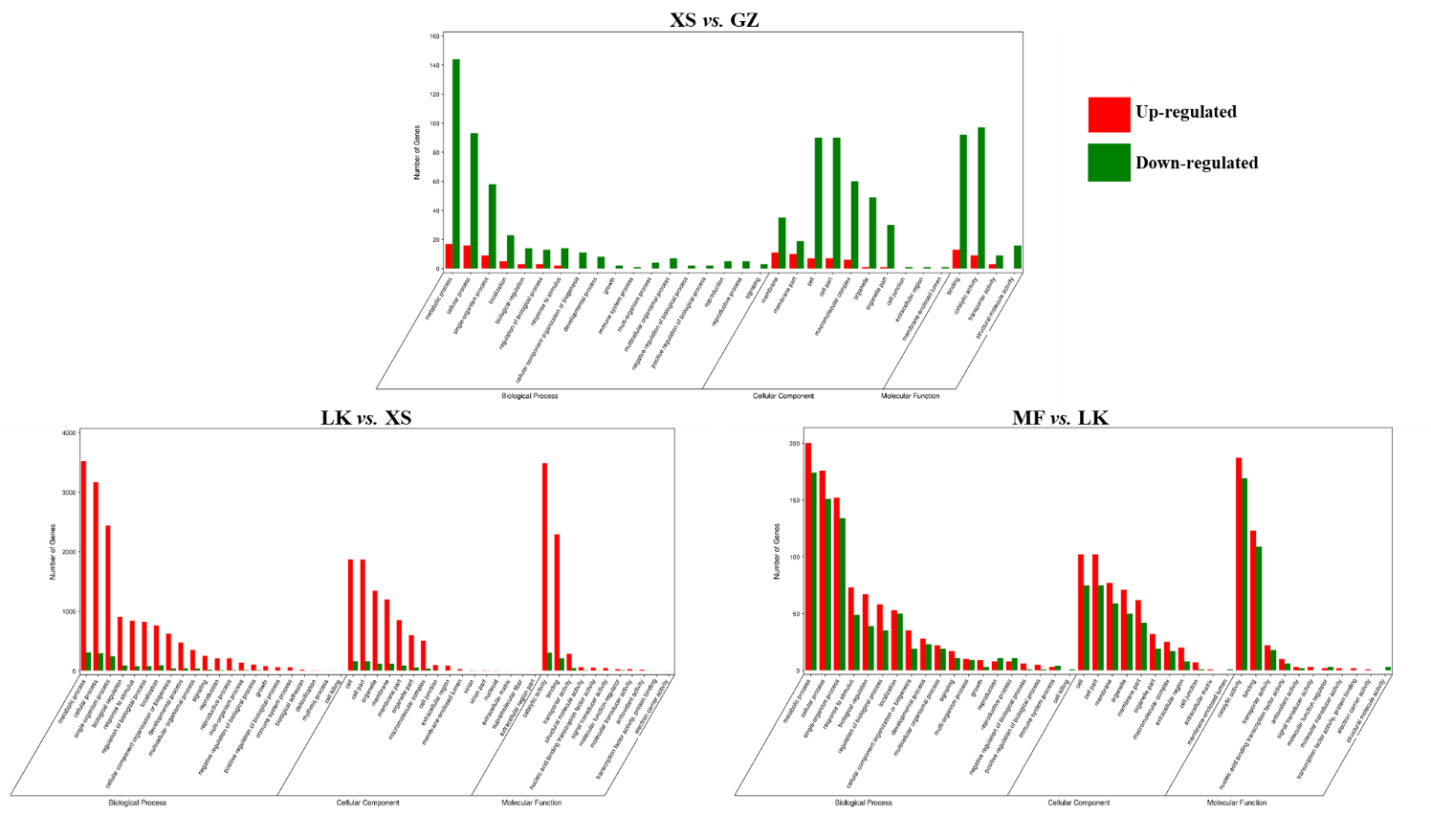


**Figure S1 GO classification of the differentially expressed genes in the continuous comparison system of *Cinnamomum migao* seed germination.**

The abscissa represents the functional classification and the ordinate represents the number of genes in the annotation.
