# Supplementary figures and images for: Transcriptome analysis of Cinnamomum migao seed germination in medicinal plants of Southwest China
Source: BMC Plant Biol. 2021 Jun 11;21:270. doi: 10.1186/s12870-021-03020-7 (PMC8194011; doi:10.1186/s12870-021-03020-7)

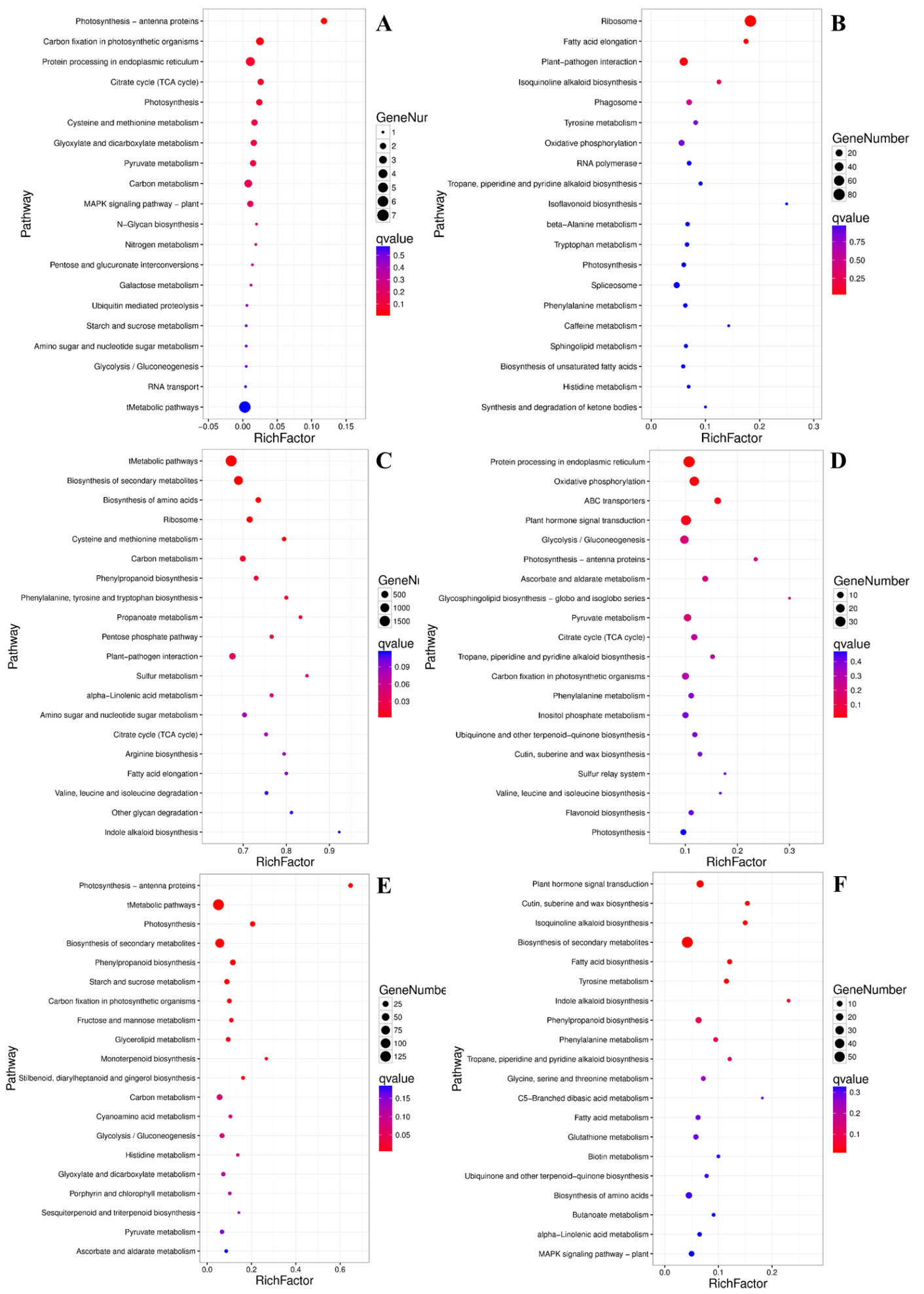


**Figure S2 KEGG enrichment of DEGs in CCS comparison group of *Cinnamomum migao* seed germination.**

Supplement: Supplementary file 2 — Figure S2 KEGG enrichment of DEGs in CCS comparison group of Cinnamomum migao seed germination. [file 12870_2021_3020_MOESM2_ESM.docx]
