# Supplementary material for: Transcriptome analysis of Cinnamomum migao seed germination in medicinal plants of Southwest China
Source: BMC Plant Biol. 2021 Jun 11;21:270. doi: 10.1186/s12870-021-03020-7 (PMC8194011; doi:10.1186/s12870-021-03020-7)
Supplement: Supplementary file 3 — Figure S3 Cluster analysis of differentiallyexpressed genes related to the lipid metabolism pathway in the four germinationstages of Cinnamomum migao seeds.A, B. Cluster analysis of differentially expressed genes related to glycerolmetabolism pathway. C. Cluster analysis of differentially expressed genesrelated to fatty acid degradation pathway. [file 12870_2021_3020_MOESM3_ESM.docx]

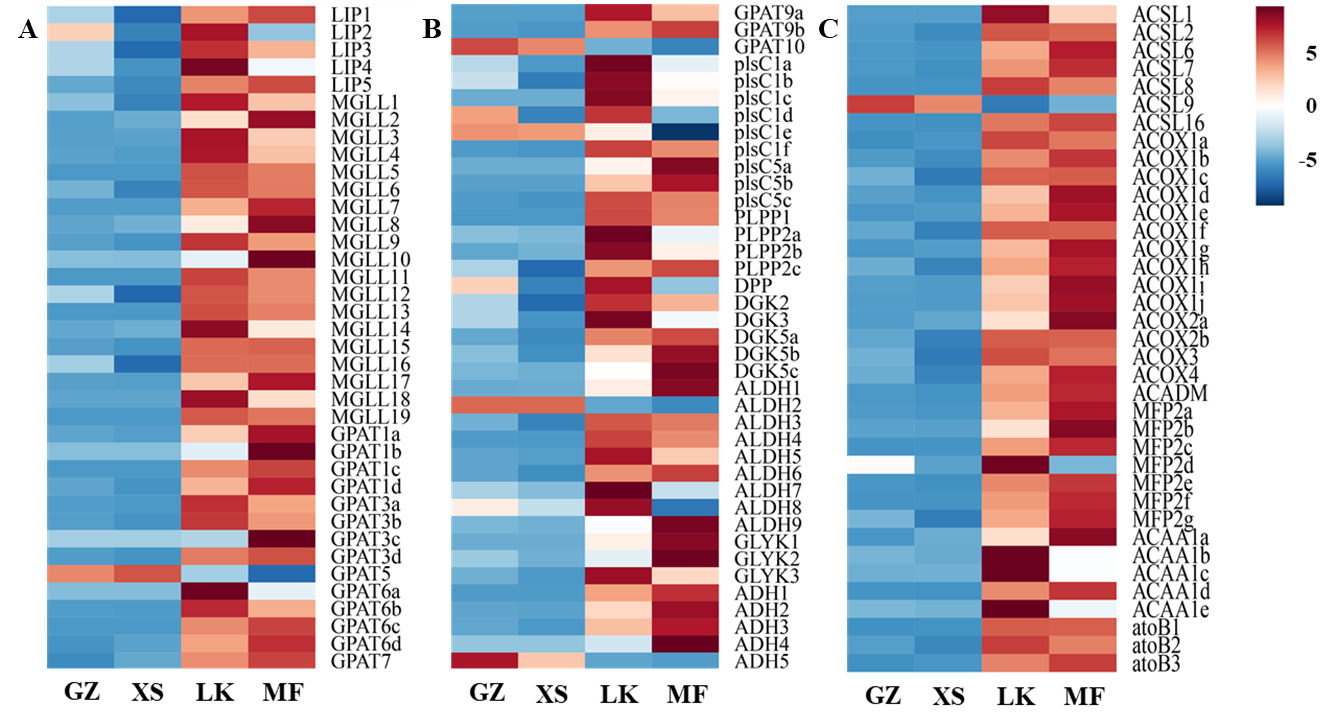


**Figure S3 Cluster analysis of differentially expressed genes related to the lipid metabolism pathway in the four germination stages of *Cinnamomum migao* seeds.**

A, B. Cluster analysis of differentially expressed genes related to glycerol metabolism pathway. C. Cluster analysis of differentially expressed genes related to fatty acid degradation pathway.
