# Supplementary material for: Transcriptome analysis of Cinnamomum migao seed germination in medicinal plants of Southwest China
Source: BMC Plant Biol. 2021 Jun 11;21:270. doi: 10.1186/s12870-021-03020-7 (PMC8194011; doi:10.1186/s12870-021-03020-7)
Supplement: Supplementary file 4 — Figure S4 Clusteranalysis of differentially expressed genes related tothe starch and sucrose metabolism pathways in the four germination stages of Cinnamomum migao seeds. [file 12870_2021_3020_MOESM4_ESM.docx]

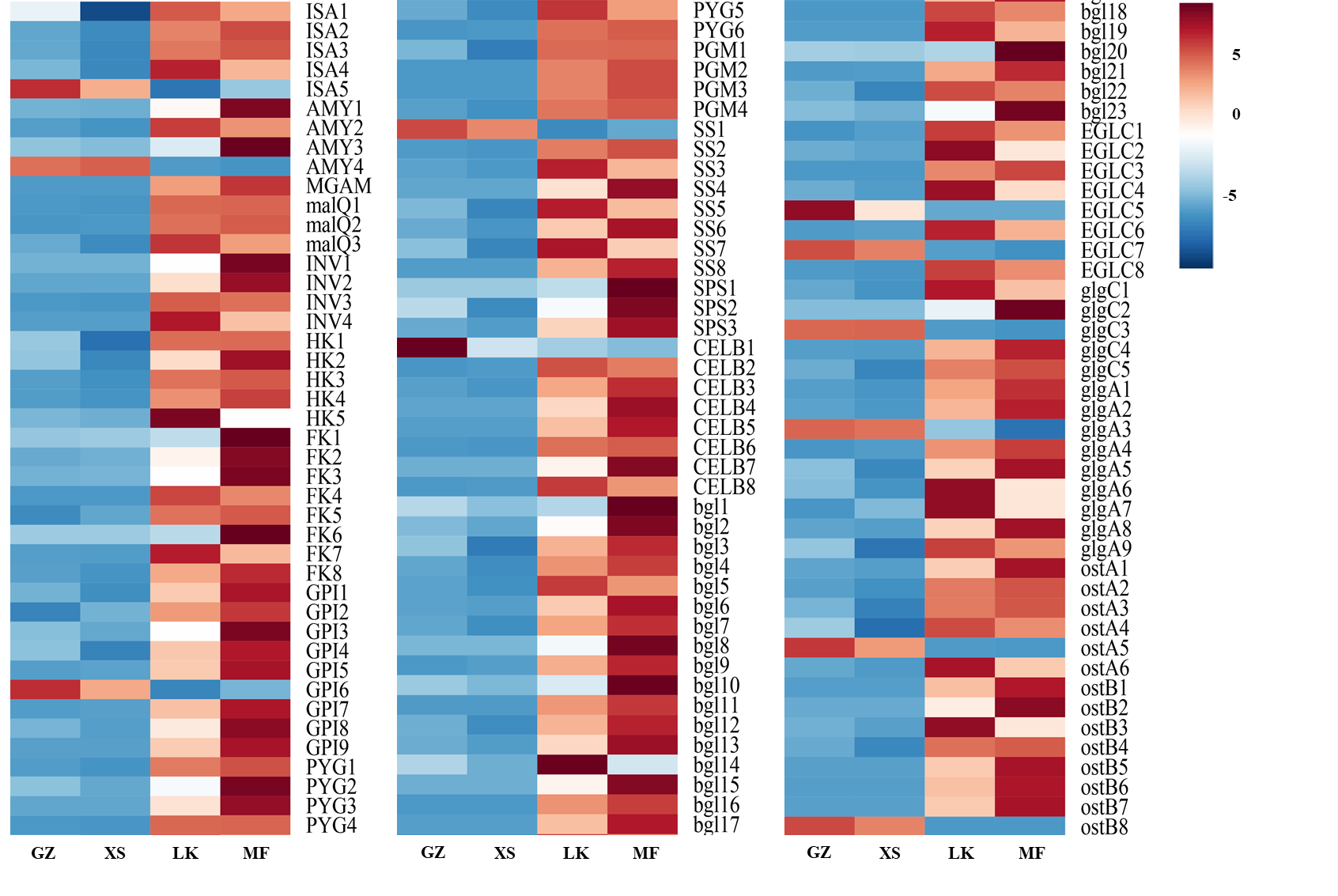


**Figure S4 Cluster analysis of differentially expressed genes related to the starch and sucrose metabolism pathways in the four germination stages of *Cinnamomum migao* seeds.**
