# Supplementary material for: Transcriptome analysis of Cinnamomum migao seed germination in medicinal plants of Southwest China
Source: BMC Plant Biol. 2021 Jun 11;21:270. doi: 10.1186/s12870-021-03020-7 (PMC8194011; doi:10.1186/s12870-021-03020-7)
Supplement: Supplementary file 6 — Table S1 Statistics ofdifferent germination stages sequencing data of Cinnamomum migao seeds. a. Before filter reads number, b. Afterfilter high quality reads number, c. After filter clean data bases, d. Afterfilter high-quality clean data bases. [file 12870_2021_3020_MOESM6_ESM.docx]

**Table S1 Statistics of different germination stages sequencing data of *Cinnamomum migao* seeds**

| **Sample** | **Clean Reads Num^a^** | **HQ Clean Reads Num^b^** | **Clean Data (bp)^c^** | **HQ Clean Data (Q30)^d^** |
| --- | --- | --- | --- | --- |
| GZ-1 | 7025923500 | 45493112 | 7025923500 | 6346659239 |
| GZ-2 | 6696058800 | 43369522 | 6696058800 | 6072844573 |
| GZ-3 | 8638104600 | 56282494 | 8638104600 | 7906591127 |
| XS-1 | 8352609600 | 55235450 | 8352609600 | 7952625140 |
| XS-2 | 8836597200 | 58447244 | 8836597200 | 8409200940 |
| XS-3 | 8764629600 | 57983422 | 8764629600 | 8353219432 |
| LK-1 | 8756202000 | 56889434 | 8756202000 | 7988308105 |
| LK-2 | 7172141100 | 46552926 | 7172141100 | 6550239049 |
| LK-3 | 9392530200 | 60978086 | 9392530200 | 8549886330 |
| MF-1 | 8331901500 | 54157930 | 8331901500 | 7580542023 |
| MF-2 | 6567141600 | 42533782 | 6567141600 | 5956790639 |
| MF-3 | 7115678400 | 46135628 | 7115678400 | 6485367679 |

a. Before filter reads number, b. After filter high quality reads number, c. After filter clean data bases, d. After filter high-quality clean data bases.
