# Supplementary material for: Transcriptome analysis of Cinnamomum migao seed germination in medicinal plants of Southwest China
Source: BMC Plant Biol. 2021 Jun 11;21:270. doi: 10.1186/s12870-021-03020-7 (PMC8194011; doi:10.1186/s12870-021-03020-7)
Supplement: Supplementary file 7 — Table S2 Source andtarget pathways of the pathway-net during Cinnamomummigao seed germination. Degree means the number of relationships that onepathway has with other pathways, the value of degree represents theirsignificance. Indegree is the target pathway, outdegree is the source pathway. [file 12870_2021_3020_MOESM7_ESM.docx]

**Table S2 Source and target pathways of the pathway-net during *Cinnamomum migao* seed germination**

| **Pathway name** | **Degree** | **Indegree** | **Outdegree** |
| --- | --- | --- | --- |
| citrate cycle(TCA cycle) | 28 | 16 | 12 |
| Glycolysis/Gluconegenesis | 23 | 12 | 11 |
| Pyruvate metabolism | 19 | 9 | 10 |
| Propanoate metabolism | 11 | 7 | 4 |
| Fructose and mannose metabolism | 11 | 4 | 7 |
| Pentose phosphate pathway (PPP) | 10 | 4 | 6 |
| Glycerophospholipid metabolism | 10 | 4 | 6 |
| Arginine and proline metabolism | 10 | 3 | 7 |
| Tyrosine metabolism | 8 | 2 | 6 |
| Starch and sucrose metabolism | 8 | 3 | 5 |
| Ascorbate and aldarate metabolism | 8 | 4 | 4 |
| Glycerolipid metabolism | 7 | 3 | 4 |
| Pentose and glucuronate interconversions | 6 | 4 | 2 |
| Glyoxylate and dicarboxylate metabolism | 6 | 4 | 2 |
| Galactose metabolism | 6 | 1 | 5 |
| Tryptophan metabolism | 5 | 1 | 4 |
| Synthesis and degradation of ketone bodies | 5 | 1 | 4 |
| Phenylpropanoid biosynthesis | 5 | 2 | 3 |
| Alanine, aspartate and glutamate metabolism | 5 | 2 | 3 |
| Lysine degradation | 4 | 3 | 1 |
| Glycine, serine and threonine metabolism | 4 | 0 | 4 |
| Cysteine and methionine metabolism | 4 | 2 | 2 |
| Ascorbate and aldarate, glutamate metabolism | 4 | 3 | 1 |
| Amino sugar and nucleotide sugar metabolism | 4 | 4 | 0 |
| Phenylalanine, tyrosine and tryptophan biosynthesis | 3 | 0 | 3 |
| Galactose metabolism | 3 | 1 | 2 |
| Arginine biosynthesis | 3 | 1 | 2 |
| Amino sugar and nucleotide sugar metabolism | 3 | 0 | 3 |
| Valine, leucine and isoleucine degradation | 2 | 0 | 2 |
| propanoate metabolism | 2 | 2 | 0 |

Degree means the number of relationships that one pathway has with other pathways, the value of degree represents their significance. Indegree is the target pathway, outdegree is the source pathway.
