# Supplementary material for: Transcriptome analysis of Cinnamomum migao seed germination in medicinal plants of Southwest China
Source: BMC Plant Biol. 2021 Jun 11;21:270. doi: 10.1186/s12870-021-03020-7 (PMC8194011; doi:10.1186/s12870-021-03020-7)
Supplement: Supplementary file 8 — Table S3 Sequences ofprimers used for qRT-PCR. [file 12870_2021_3020_MOESM8_ESM.docx]

**Table S3 Sequences of primers used for qRT-PCR**

| **Unigene ID** | **Primer sequence (5'->3')** | **Functional annotation** |
| --- | --- | --- |
| >Unigene0010885 | F:TGGCGACGCAAAGATCAAAT | ATCB |
|  | R:CACATCCTCACCAACCCACG |  |
| >Unigene0011483 | F:GCCCAGTACAAGCCAGAACCAT | UBQ |
|  | R:CCTTTTGAGAAATCCGACCCA |  |
| >Unigene0038457 | F:CATCTTAACCAATCCTGCATCAC | TUB |
|  | R:TTATCATCACGAACCACCCTG |  |
| >Unigene0000183 | F:CATTGCCTTTCCACAGTGAGACC | SS |
|  | R:CCAAGCCACTGGGACAGAATC |  |
| >Unigene0009738 | F:GCGGTCGCCATTACAAAGC | FK2 |
|  | R:TGAGAAGCATATCGGCACTCG |  |
| >Unigene0013221 | F:GAAGGAGGGCATCTTGAGCAT | FK3 |
|  | R:ACCACAGGGACATCACCACATC |  |
| >Unigene0041154 | F:CATCAACAATGGCGAAAGACG | MDH1 |
|  | R:GCATGTGCAGAATCACAGGCT |  |
| >Unigene0044781 | S:ACGCAAGAAGGAGGGACGGA | MDH2 |
|  | R:TCAGAAAGGGAACCCAACCCAAGAAC |  |
| >Unigene0033391 | F:CAGATGGAAACACCAAGGACG | PYL |
|  | R:GGCAATCCCACTGTATCTCACC |  |
| >Unigene0030685 | F:GGCTCCTGCTTCCACCTCTT | ABF |
|  | R:CGCCATAACCACGCCCGATAAA |  |
| >Unigene0053879 | F:GAATAGGACTTGCTGGTCTCGC | PGD |
|  | R:TGGGGTCATGGAATCCAAATAG |  |
| >Unigene0055678 | F:TTGTTAGCACAGGAACCACCAC | PK |
|  | R:ATACCCAGAACTCGCCGTCA |  |
| >Unigene0049470 | F:TGCTGTTTTCGGGCTTGG | ADH |
|  | R:TGGTTTGTCATGGTCTTTTGGGT |  |
| >Unigene0006719 | F:TCAAGTGCGGAGGAATGGAC | HK |
|  | R:GATCGTGAGGGAGTTCGAGGA |  |
| >Unigene0004288 | S:ATCGCCATGCTCATACCGC | PPFK |
|  | R:ACCAAATCAACTCCGTCCCTCA |  |
